# A Competition between Hydrogen, Stacking, and Halogen Bonding in *N*-(4-((3-Methyl-1,4-dioxo-1,4-dihydronaphthalen-2-yl)selanyl)phenyl)acetamide: Structure, Hirshfeld Surface Analysis, 3D Energy Framework Approach, and DFT Calculation

**DOI:** 10.3390/ijms23052716

**Published:** 2022-02-28

**Authors:** Mohamed Gouda, Hela Ferjani, Hany M. Abd El-Lateef, Mai M. Khalaf, Saad Shaaban, Tarek A. Yousef

**Affiliations:** 1 Department of Chemistry, College of Science, King Faisal University, P.O. Box 380, Al-Ahsa 31982, Saudi Arabia; mgoudaam@kfu.edu.sa (M.G.); hmahmed@kfu.edu.sa (H.M.A.E.-L.); mmkali@kfu.edu.sa (M.M.K.); 2 Department of Chemistry, College of Science, IMSIU (Imam Mohammad Ibn Saud Islamic University), P.O. Box 5701, Riyadh 11623, Saudi Arabia; hhferjani@imamu.edu.sa; 3 Chemistry Department, Faculty of Science, Sohag University, P.O. Box 82524, Sohag 82524, Egypt; 4 Department of Chemistry, Organic Chemistry Division, College of Science, Mansoura University, P.O. Box 11432, Mansoura 11001, Egypt; 5 Toxic and Narcotic Drug, Forensic Medicine Department, Mansoura Laboratory, Medicolegal Organization, Ministry of Justice, P.O. Box 12432, Cairo 11435, Egypt

**Keywords:** crystal structure, organoselenium, antioxidant, cytoprotective, Hirshfeld surface analysis, DFT calculations, 3D energy framework

## Abstract

*N*-(4-((3-Methyl-1,4-dioxo-1,4-dihydronaphthalen-2-yl)selanyl)phenyl)acetamide (5), C_19_H_15_NO_3_Se, was prepared in two steps from 4,4′-diselanediyldianiline (3) via reduction and subsequent nucleophilic reaction with 2-methyl-3-bromo-1,4-naphthalenedione, followed by acetylation with acetic anhydride. The cytotoxicity was estimated against 158N and 158JP oligodendrocytes and the redox profile was also evaluated using different in vitro assays. The technique of single-crystal X-ray diffraction is used to confirm the structure of compound 5. The enantiopure 5 crystallizes in space group P21 with Flack parameter 0.017 (8), exhibiting a chiral layered absolute structure. Molecular structural studies showed that the crystal structure is foremost stabilized by N-H···O and relatively weak C-H···O contacts between molecules, and additionally stabilized by weak C-H···π and Se···N interactions. Hirshfeld surface analysis is used to quantitatively investigate the noncovalent interactions that stabilize crystal packing. Framework energy diagrams were used to graphically represent the stabilizing interaction energies for crystal packing. The analysis of the energy framework shows that the interactions energies of and C-H···π and C-O···π are primarily dispersive and are the crystal’s main important forces. Density functional theory (DFT) calculations were used to determine the compound’s stability, chemical reactivity, and other parameters by determining the HOMO-LUMO energy differences. The determination of its optimized surface of the molecular electrostatic potential (MEP) was also carried out. This study was conducted to demonstrate both the electron-rich and electron-poor sites.

## 1. Introduction

Selenium (Se) is a substantial micronutrient pivotal for the normal function of the human body [1,2,3,4]. It is found in foods (e.g., tuna, sardines beans, spinach, Brazil nuts) as well as in the structure of different proteins, i.e., selenoproteins [3,4]. The latter is crucial for the chemoprotection of cells from infection, inflammation, and oxidative damage [5,6,7,8,9,10]. Importantly, the body–Se balance is fundamental for the maintenance of the immune system’s regular function and antimicrobial defense [7,8,10]. On the other hand, the imbalance of Se levels is associated with different diseases’ development, including autoimmune, heart, and cancer diseases [11,12,13,14,15,16,17,18]. Significantly, Se boosting was accompanied by the chemoprevention of various tumors (e.g., colon, breast, liver) as well as the management of rheumatoid arthritis [16,17]. In general, inorganic Se agents are generally less pharmacologically active than organoselenium compounds, which, in turn, have enhanced bioavailability owing to their superior amphiphilicity and pharmacodynamic properties [19,20,21,22,23,24,25,26]. Several key enzymes possess core Se responsible for their interesting redox potential, such as the glutathione peroxidase (GPx) and thioredoxin reductases (TrxR) [27,28,29]. Within this context, organoselenium compounds mimicking the GPx showed interesting antioxidant, anti-inflammatory, and antitumor activities [27,29,30,31,32].

**Figure 1:**
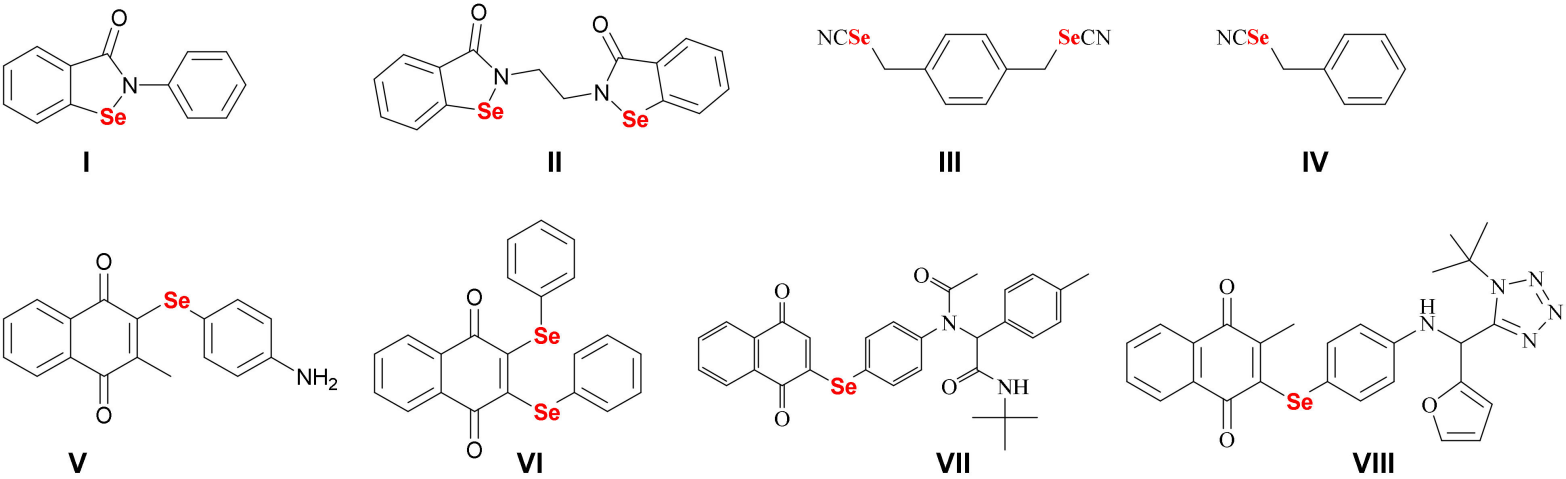
Diverse organoselenium compounds with interesting biological activities.

Ebselen (**I**) was among the first discovered Se heterocycles with potential antioxidant, anti-inflammatory, and GPx-like activities (Figure 1) [33,34,35]. Furthermore, ethaselen (**II**) has recently entered clinical trial phase I as a potential organoselenium antitumor agent with minimal cytotoxicity in vivo [36]. Moreover, several organoselenocyanates (e.g., p-xylene selenocyanate (**III**) and benzyl selenocyanate (**IV**)) exhibited interesting chemoprotective properties towards different tumor models, such as colon, lung, and liver cancers (Figure 1) [37,38,39,40,41,42,43,44]. 

Recently, we and others reported several organoselenium candidates with GPx-like and antioxidant activity even better than ebselen [40,42,45,46,47,48]. Among them, compounds containing quinone and Se motifs manifested considerable success due to their chemoprevention of carcinogenesis [38,49,50,51,52,53,54,55,56,57]. Within this context, our group reported selenoquinones (**V** and **VI**) with interesting cytotoxicity at low micromolar concentrations against different cancer types, such as breast adenocarcinoma (MCF-7), human epidermoid carcinoma, hepatocellular carcinoma (HepG2), and human kidney carcinoma cells. Indeed, selenoquinones (**VII** and **VIII**) activated the caspase-8 expression levels and downregulated the Ki-67 and Bcl-2 levels in HepG2 [49,52,53]. 

Indeed, in silico studies were used as a prediction tool to evaluate the antioxidant, cytotoxicity, chemopreventive, and pro-apoptotic activities of organoselenium compounds [4,5,6,41,55]. Within this context, we and others have shown a probable interaction between organoselenium compounds and TrxR protein, which was accordingly used to predict their antitumor activities [7,9,28,41,55]. Furthermore, 3D similarity studies disclosed an analogous shape and electrostatic pattern to ebselen, thereby proposing similar GPx-like properties [9,30,31,55]. Moreover, the ebselen underlying antiviral (e.g., coronavirus) activity was theoretically attributed to the inhibition of the SARS-CoV-2 proteases via interaction with the viruses M^pro^ and PL^pro^ [5,7,9,11].

Additionally, we are not only interested in the synthesis and biological evaluation of Se-based quinones but also in the investigation of their crystal structure elucidation. Herein, we report the crystal structure of compound **5**. The cytotoxicity was estimated against 158 N and 158 JP oligodendrocytes and the redox profile was also evaluated using different in vitro assays.

In crystal packing, the nature of intermolecular interactions was characterized through the Hirshfeld surface analysis description. In addition, DFT calculations were employed to optimize the structure of compound **5** in its isolated state. Moreover, Frontier molecular orbitals (FMO) and MEP mapping studies were achieved to identify global reactivity descriptors and the complementary interacting sites in compound **5**. 

## 2. Material and Methods

### 2.1. Synthesis of Organoselenium Compound ***5***

**Scheme 1:**
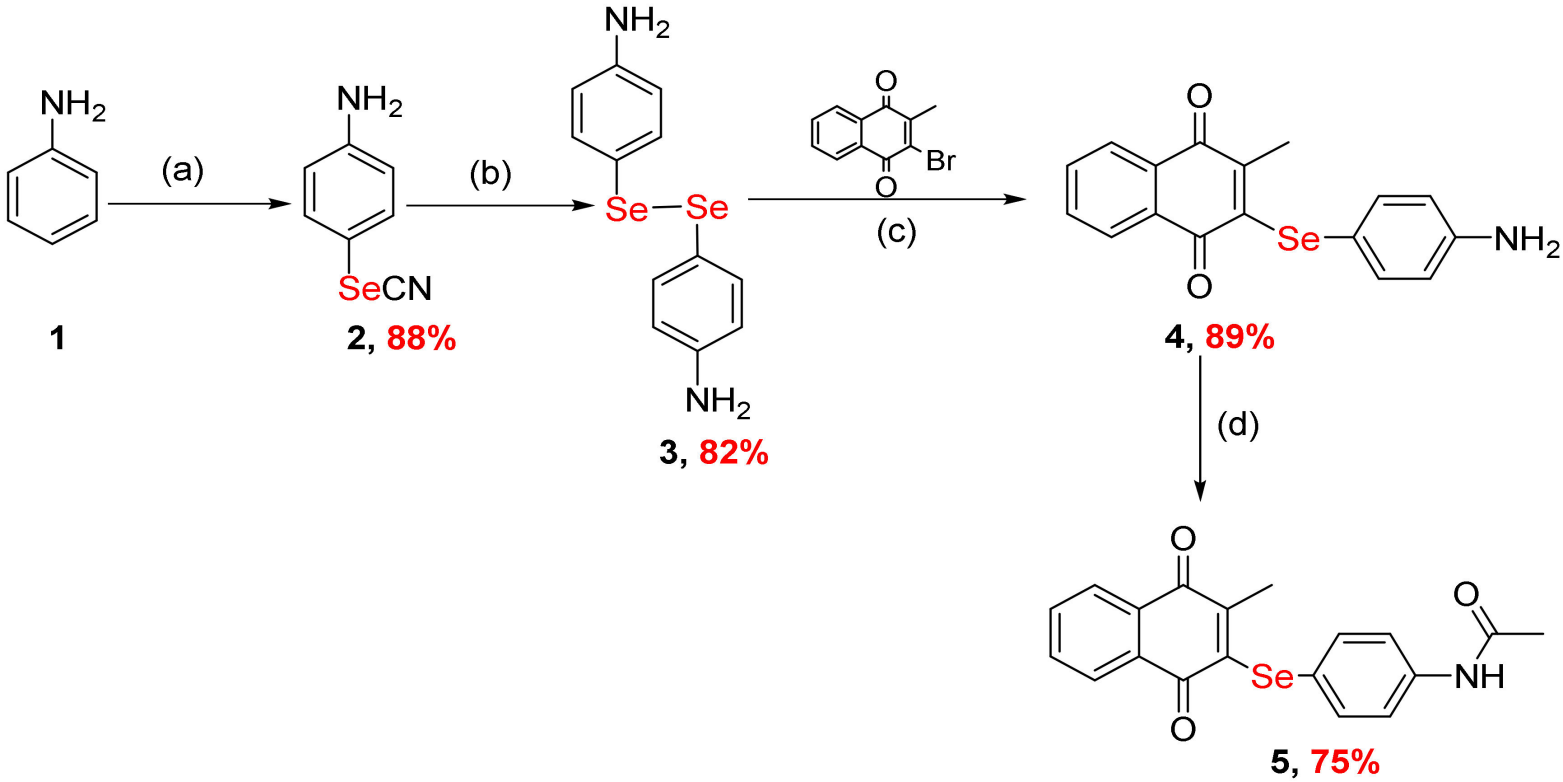
Synthesis of compound **5**. Reagents and conditions: (**a**) SeO_2_ (9.6 mmol), malononitrile (4.8 mmol), DMSO (4 mL); (**b**) 4-selenocyanatoaniline 2 (2 mmol), NaOH (3 mmol), MeOH (15 mL); (**c**) 2-methyl-3-bromo-l,4-naphthoquinone (4.4 mmol), EtOAc: water (1:1), NaBH_4_ (6 mmol), Aliquat 336 (5% mol); (**d**) acetic anhydride, 3 h, 50 °C.

The synthesis of compound **5** was conducted from 4,4′-diselanediyldianiline (**3**) and 2-methyl-3-bromo-l,4-naphthoquinon as presented in Scheme 1 [54,58].

### 2.2. Biological Evaluation

The cytotoxicity was estimated by the MTT assay against different oligodendrocytes, such as 158 JP and 158 N cells, and 7-ketocholesterol was the control [54,58]. Different in vitro assays were used to estimate the redox activity, including the dihydroethidium (DHE), 2′,7′-dichlorodihydrofluorescein diacetate (H2-DCFDA), 2,2′-azinobis-3-ethylbenzothioazoline-6-sulphonate (ABTS), 2,2-diphenyl-2-picrylhydrazyl (DPPH), and the bleomycin-induced DNA damage assays [54,58].

### 2.3. Crystal Structure Measurement

X-ray data were estimated at 115 K from a single crystal of compound **5** on a Bruker diffractometer (APEX-II CCD) using a single wavelength (λ = 0.71073). X-ray MoK_α_. SAINT [59,60] and SADABS [61] were used to reveal the multi-scan absorption correction and Data reduction. Olex2 [62] was used to solve the structure together with other programs, such as the SHELXT. Additionally, refinement of the structure was implemented with the 2018/3 package of SHELXL [63]. The hydrogens were fixed in their computed places and a riding model was used for the refinement with hydrogen lengths of 0.88 Å (NH) and 0.95–0.98 Å (CH). Displacement of the isotropic parameters was placed to 1.2 (CH, NH) or 1.5 (CH3) times U_eq_ of the parent atom. With riding coordinates, aromatic/amide H has been refined as N(H), C5(H5), C6(H6), C7(H7), C8(H8), C13(H13), C14(H14), C16(H16), and C17(H17). Idealized CH3 was refined into rotating group C11(H11A,H11B,H11C) and C19(H19A,H19B,H19C). The refinement of the twin data was made with a 0.983(8) scale factor, since this resolved compound has an absolute structure. The proof for the chirality is also given from the Flack parameter [64], which is 0.017(8). PLATON was used to check the results of the X-ray analysis [65]. Mercury 4.0 software was utilized for the single-crystal graphical representation. The relevant crystallographic information for compound **5** is tabulated in Table 1 [66].

### 2.4. Theoretical Calculations 

The Gaussian 09 program was used for all the calculations presented in this work [67]. First, the geometries of the compound considered were entirely optimized at the 6-311+G(d,p) level [68]. In the literature, the B3LYP functional is widely used and leads to dependable results regarding the organic compounds’ ground state properties [69]. The calculations for normal modes of vibration were performed using optimized geometries, which led to real frequencies that indicated that these geometries were minimums on the potential energy surfaces. We also used the energy quantities to research and confirm the stability of these compounds. The results obtained were compared with the experimental data. Empirical corrections for dispersion were evaluated using DFT-D corrections [70], which necessarily deepens the interaction well and is an essential correction to the DFT energy of this compound.

## 3. Results and Discussion

### 3.1. Design and Synthesis of Compound ***5***

Selenium dioxide and malonitrile are being used as an efficient selenocyanating medium for several aromatic hydrocarbons, such as aniline [59]. In this case, the corresponding 4-selenocyanatoaniline (**2**) was obtained with a good yield (88%). The 4,4′-Diselanediyldianiline (**3**) was prepared with an 82% yield via the hydrolysis of 4-selenocyanatoaniline (**2**) using strong alkali (e.g., NaOH). The 4,4′-Diselanediyldianiline (**3**) was used for the synthesis of diverse organoselenium compounds by the reduction of the diselenide bond followed by a nucleophilic reaction with different halogenated compounds. Within this context, the reduction of diselenide compound **3** with NaBH_4_ and the succeeding treatment with 2-methyl-3-bromo-l,4-naphthoquinone in a two-phase solvent (ethyl acetate and water 1:1) system and 5% mol Aliquat 336 as the phase transfer agent afforded compound **4** an 89% yield in Scheme 1. The reaction of compound **4** with acetic anhydride furnished compound **5** with a 75% yield (Scheme 1) [54,58].

### 3.2. Biology

Oligodendrocytes are pivotal for neuronal signal transmission and the axon integrity; however, they are prone to damage by reactive nitrogen and oxygen species [49,50,52,53,54]. Several organoselenium agents were reported as having potential cytoprotective and antioxidant activities [50,53,54]. Compound **4** demonstrated interesting cytotoxic activity against HepG2 (IC_50_ = 0.9 μM) and MCF-7 (IC_50_ = 14.7 μM) as well as 158N (IC_50_ = 27 μM) oligodendrocytes. Acetanilide-based selenoquinone **5** also exhibited potential antiproliferative activity against the 158 N (IC_50_ = 11 μM) and 158 JP (IC_50_ = 0.02 μM) oligodendrocytes (see Supplementary Materials) [54,58].

The levels of ROS were monitored with the H2-DCFDA and DHE assays using flow cytometry. Briefly, 158 N cells were mixed with serial concentrations (0–50 μM) of compound **4** and compound **5** using Vitamin E as the control.

In the H2-DCFDA assay, compound **4** showed a pronounced pro-oxidant activity. On the other hand, compound **5** was able to decrease the H2-DCF intensity, thus diminishing the ROS level at 10 μM compared to vitamin E. In the DHE assay, compound **4** was able to promote the production of O_2_^−^ in 158 N cells [54,58].

Moreover, compound **4** and compound **5** exhibited moderate GPx-like activity. Additionally, bleomycin-induced DNA damage, DPPH, and ABTS in vitro assays were employed to estimate compound **4** and **5’s** radical scavenging activities. Compounds **4** and **5** manifested good pro-oxidant properties (up to 60% compared to vitamin C). Interestingly, these data were in good agreement with that obtained from the DHE and H2DCF assays [54,58].

### 3.3. Analysis of the Molecular Packing 

**Figure 2:**
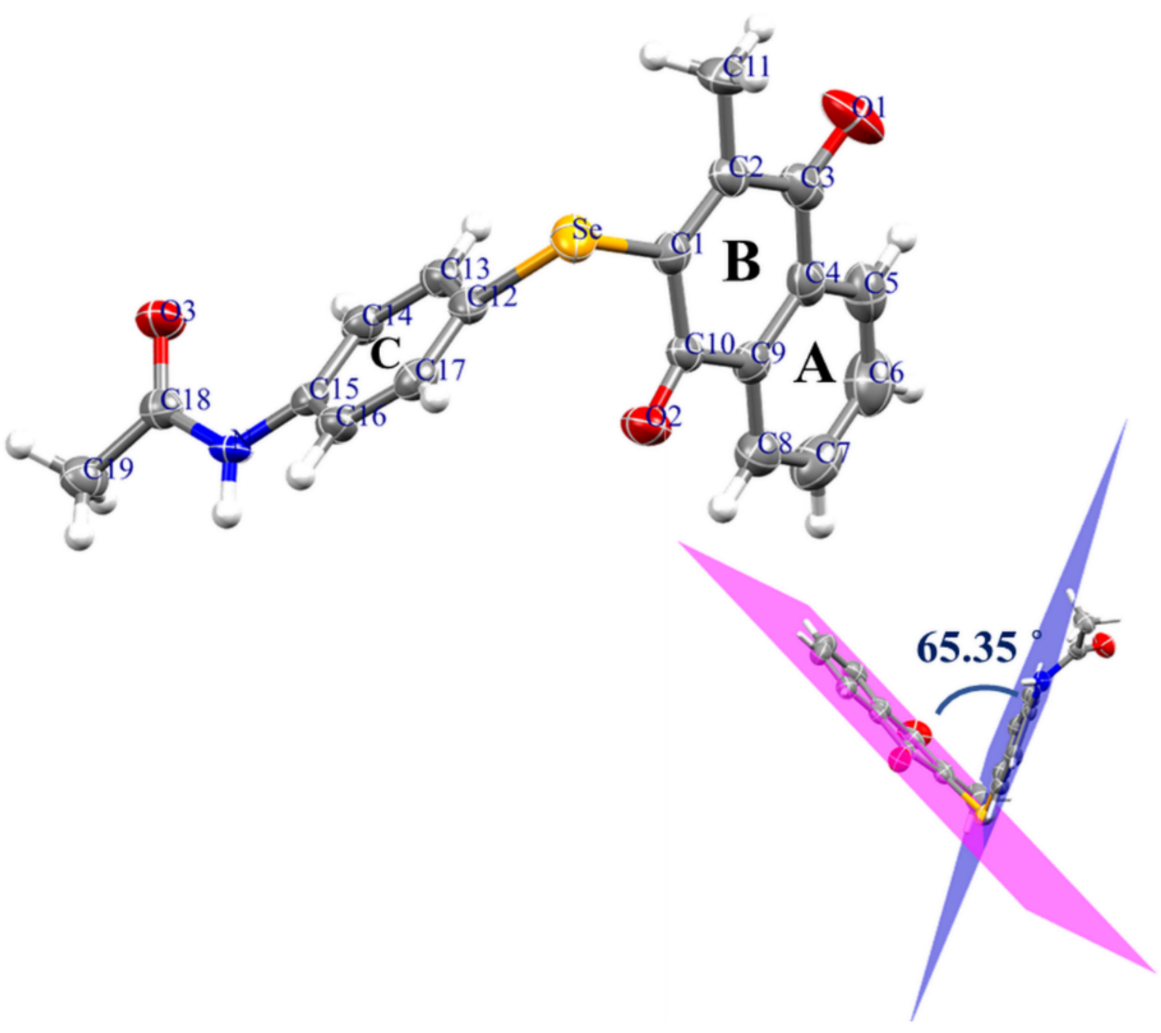
Molecular structure and atom-labeling scheme for structure compound **5**. Benzene ring (**A**) and phenyl ring (**B**), and another phenyl ring (**C**). For all atoms, displacement ellipsoids are drawn at the 80% probability level, except for H atoms, which are drawn at the 20% probability level.

**Figure 3:**
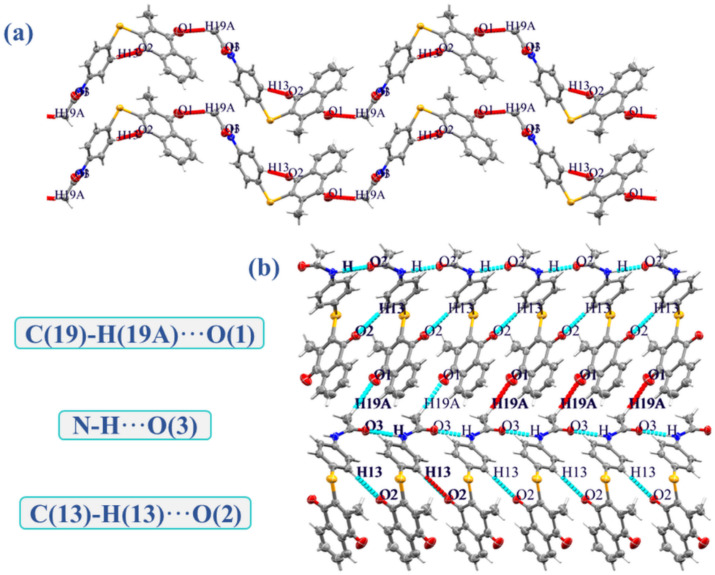
C-H···O and N-H···O Hydrogen bond interactions (**a**) along *a* axis and (**b**) along *c* axis in compound **5**.

**Figure 4:**
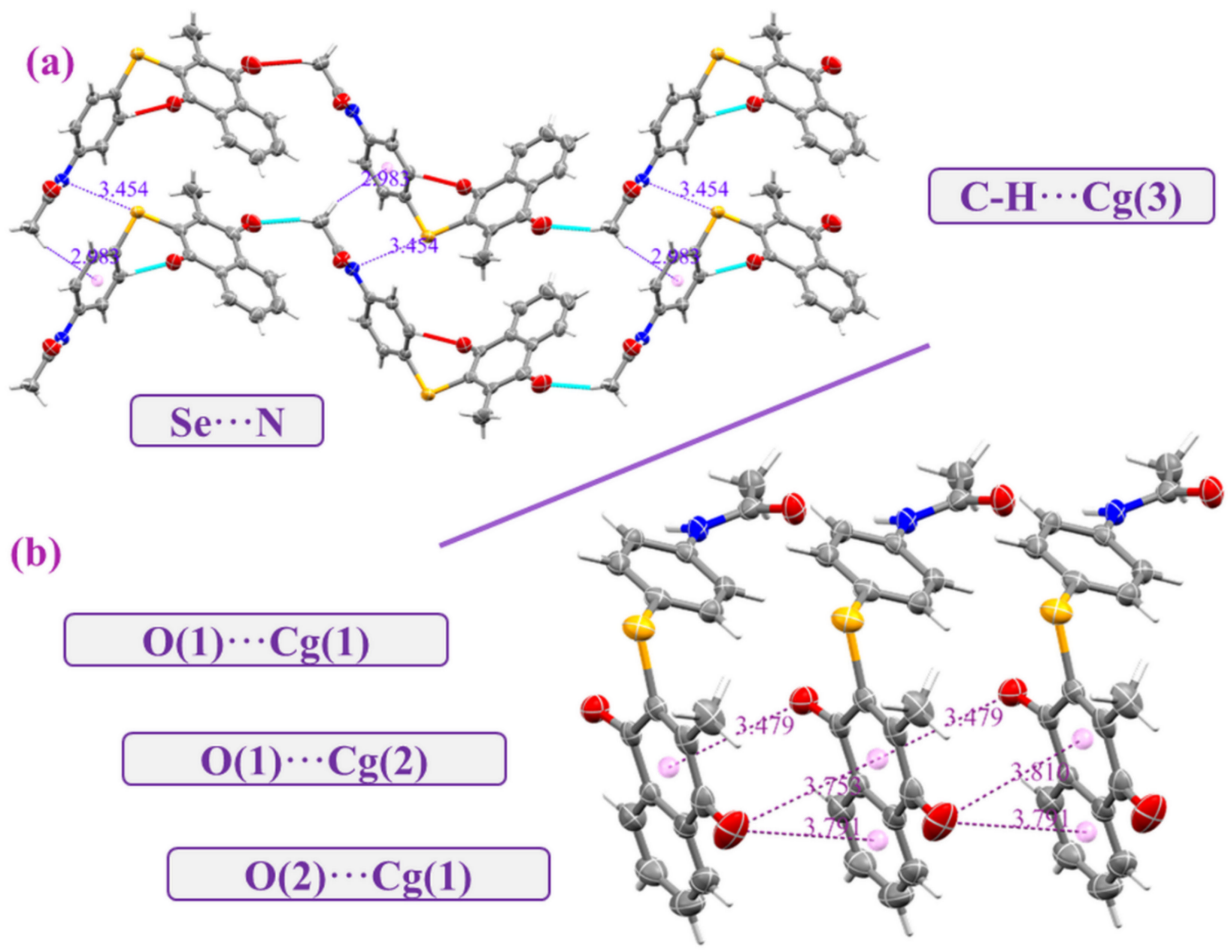
(**a**) C-H···πand Se···N (**b**) C-O···π interactions in compound **5**.

Compound **5** crystallizes in the monoclinic system with the chiral space group *P2_1_*. Its asymmetric unit (Figure 2) is composed of one chiral molecule. The Flack parameter of the absolute configuration of compound **5** is found + 0.017(8), indicating that the configuration is R. The FLACK parameter with a value of 0 indicates the correct structure and 1 indicates the inverted structure. The main body of compound **5’s** structure consists of two nearly coplanar fused rings, a benzene ring (A) and phenyl ring (B), and another phenyl ring (C) attached to the Se atom—see Figure 2. The two planes composed by (A, B) and C rings are twisted to each other by 65.35°. The C1-Se–C12 angle is 97.36 (11) Å (Table 2), which is like the values reported in the literature for the three related compounds [71,72,73]. The Se-C1 and Se-C12 bond lengths are found to be 1.916(3) Å and 1.924(3) Å, respectively, and may be regarded as normal (Table 2) [74,75]. The PLATON analysis suggests that these molecules are bonded together by several types of noncovalent interactions, such as hydrogen bonds (C-H···O, N-H···O, C-H···π), C-O···π, and unusual short contacts such as Se···N contacts. The C(19)-H(19A)···O(1) and N-H···O(3) hydrogen bonds connect molecules into double layers that stack along the a and c axis (Figure 3a,b, Table 3). The double chains are also stabilized utilizing C(19)-H(19C)···Cg3 {3.7922(4) Å, Cg3= centroids C}, and Se···N (3.4541(4) Å) contacts (Figure 4a). The phenyl and benzyl rings have participated in intermolecular C(3)-O(1)···Cg(1)/Cg(2) (3.7530(4) Å and 3.7913(4) Å) and O(2)···Cg(1) (3.4784(4) Å) interactions (Figure 4b).

### 3.4. Hirshfeld Surface Analysis

**Figure 5:**
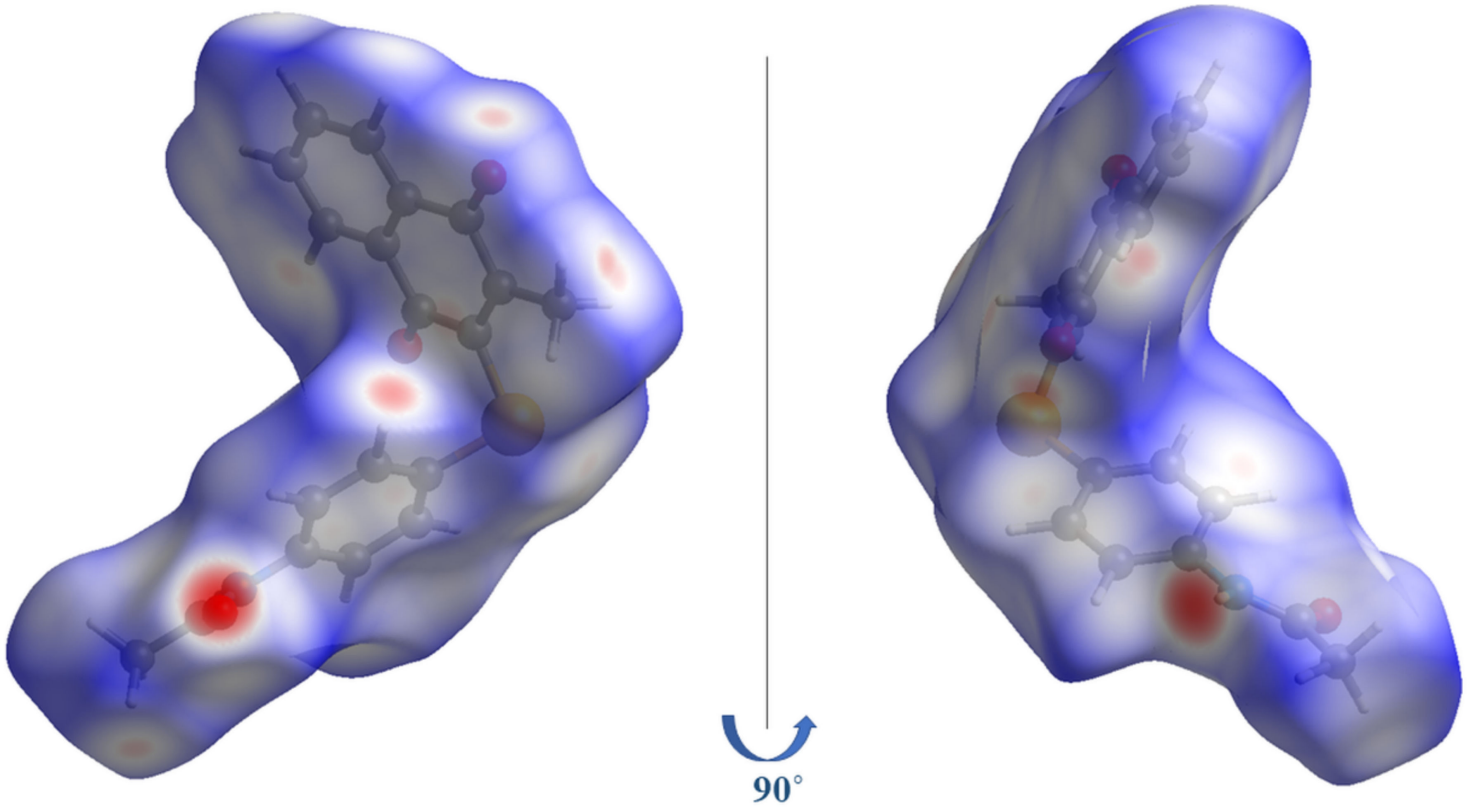
HS plotted over d_norm_ for compound **5**.

**Figure 6:**
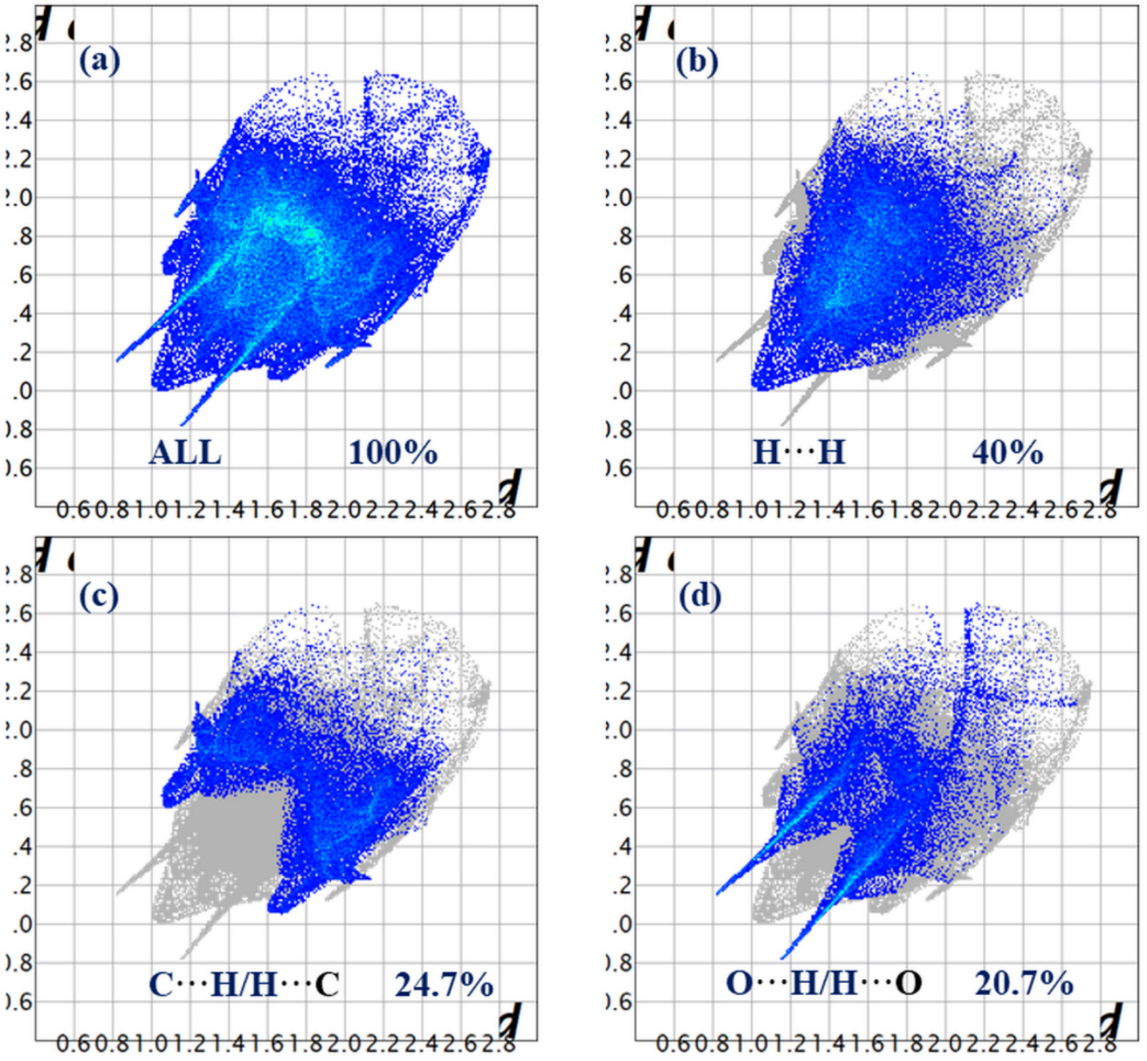
2D fingerplots for overall interactions (**a**) and significant interactions (**b**–**d**) in crystal packing of compound **5**.

**Figure 7:**
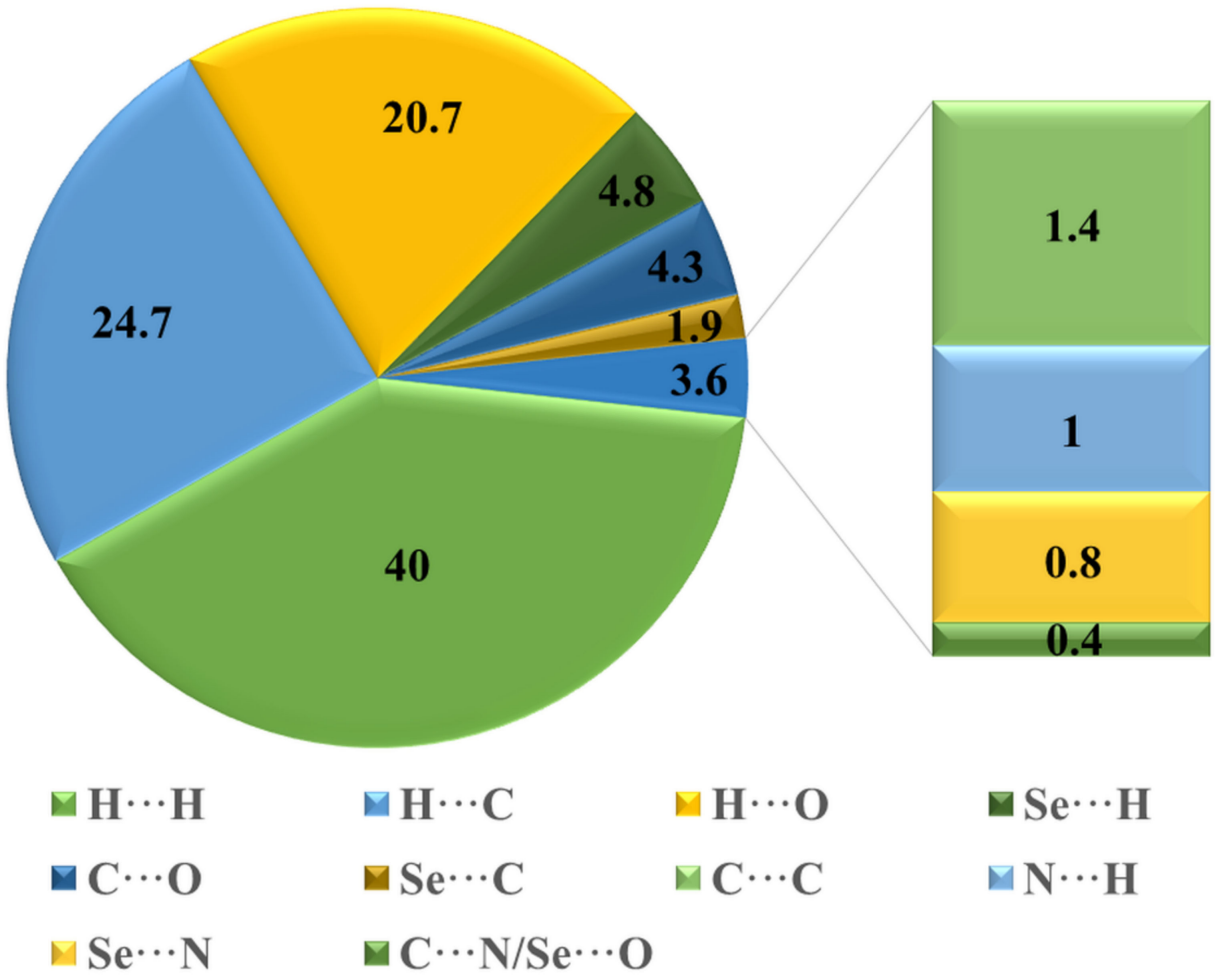
The percentage contribution of various interatomic contacts occurring in the crystal of 5.

The idea of Hirshfeld surface (HS) results come from a desire to interpret the space engaged by a molecule in a crystal to divide the crystal’s electron density into molecular fragments of electron densities. The nature of intermolecular interactions can be quantified using an HS investigation. CrystalExplorer17.5 was used to perform the HS calculations [76]. Figure 5 shows the HS plotted over d_norm_, a property with values ranging from −0.4854 (red) to 1.2838 (blue) a.u. [77]. The intermolecular contacts are visualized using color-coding. Intermolecular contacts with distances less than, equal to, and greater than van der Waal radii are shown in red, white, and blue spots, respectively. The presence of the N-H···O interaction in crystal packing is indicated by the intense red regions around the NH and O atoms of the carbonyl groups of the cation, whereas the C-H···O interaction is indicated by the pale-red spot-on HS. Additionally, two-dimensional (2D) finger-plots are employed to give quantitative data regarding the type and nature of the intermolecular interactions that are candidates in the crystal packing [78]. 2D Fingerplots are computed for each interatomic contact and the overall interactions. The reciprocal contact of individually interatomic contact is also comprised in the computation of each interatomic contact. The 2D Fingerplots for the overall interactions is shown in Figure 6a. Interatomic contacts that contribute significantly to crystal packing are represented by spikes in this graph. H···H, C···H, and O···H are the interatomic contacts that contribute the most to crystal packing, with percentage contributions of 40% (Figure 5b), 24. 7% (Figure 6c), and 20.7% (Figure 6d), respectively. Se···H, O···C, Se···C, C···C, N···H, Se···N, C···N, and Se···O are the other interatomic contacts that play a smaller role in crystal packing, with percentage contributions of 4.8%, 4.3%, 1.9%, 1.5%, 0.9%, 0.8%, 0.2% and 0.2%, respectively (Figure 7).

### 3.5. Energy Framework

**Figure 8:**
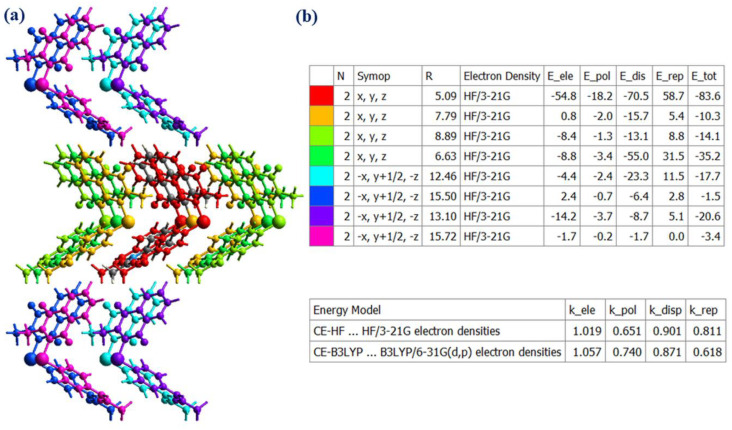
(**a**) Chemical diagram for compound **5** (**b**) the color-coded interaction mapping within 3.8 Å of the centering molecule (grey) calculated with the CE-HF…HF/3-21G model.

**Figure 9:**
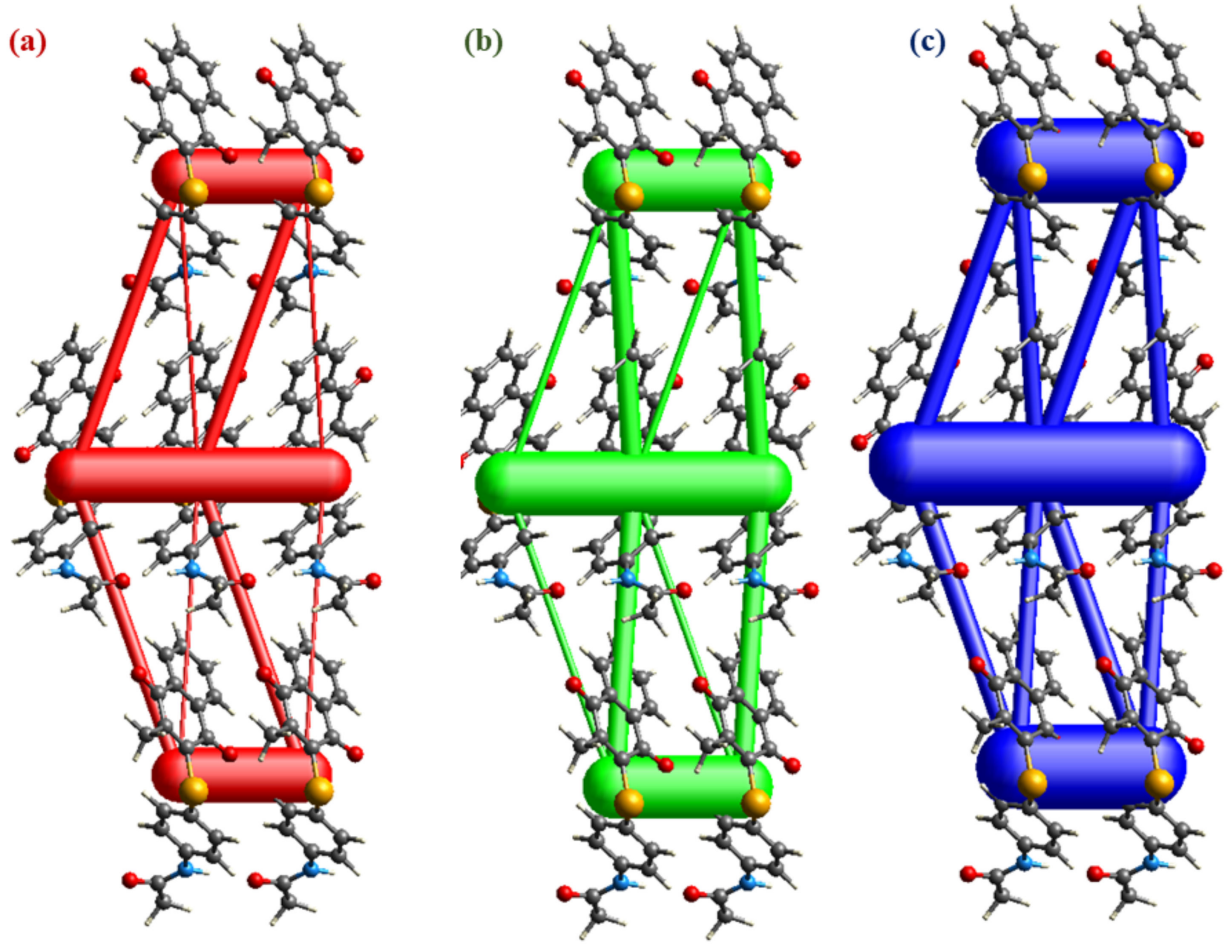
Energy framework diagram for separate electrostatic (**a**), dispersion (**b**), and the total interaction energy (**c**) of compound **5**.

Energy frameworks could be simulated using Crystal Explorer17 software. The latter is a graphical representation of individual energy components as cylinders connecting the centroids of interacting molecular pairs, with E_ele_, E_dis_, and E_tot_ color-coded in red, green, and blue, respectively, and the interaction energy’s magnitude proportional to the radius of the corresponding cylinders. Therefore, the intermolecular interaction energies for compound **5** are calculated using the CE-HF…HF/3-21G energy model presented in Crystal-Explorer [76]. Figure 8a graphically depicts the interaction energies calculations as framework energy diagrams. The components of the interaction energies (E), rotational symmetry operations concerning the reference molecule (Symop), the centroid-to-centroid distance between the reference molecule and interacting molecules (R), and the number of pair(s) of interacting molecules to the reference molecule (N) are listed in the table in Figure 8b. The results provide the total interaction energy of −186.4 kJ/mol involving the electrostatic (−89.1 kJ/mol), polarization (−31.9 kJ/mol), dispersion (−194.4 kJ/mol), and repulsion (123.8 kJ/mol). In addition, the C-H···π and C-O···π contacts highlighted in Figure 4b—between the central molecule (grey molecule) and the x, y, z symmetry-related molecule (red)—are, by far, the strongest interaction among near neighbors with an interaction energy of −83.6 kJ mol^−1^. Hydrogen bonds with C-H···O interactions energies (shamrock green) have an energy value of −35.2 kJ/mol. The total energy diagram (Figure 9c) showed a strong resemblance to the dispersion energy frameworks (Figure 9b), indicating that they play a significant role in the total forces in crystal packing.

### 3.6. DFT Calculations

#### 3.6.1. Geometric Structures

**Figure 10:**
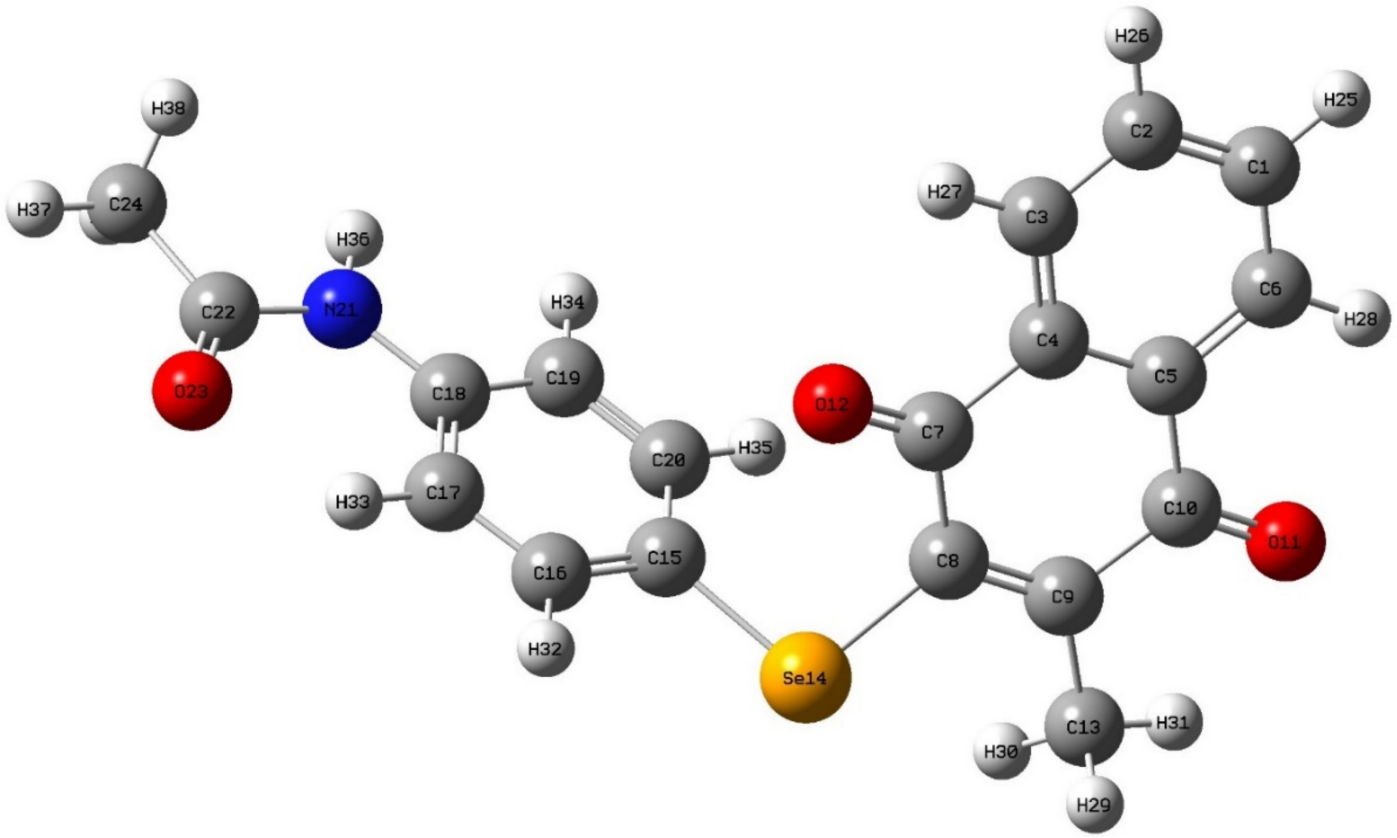
Optimized molecular structure of compound **5**.

**Figure 11:**
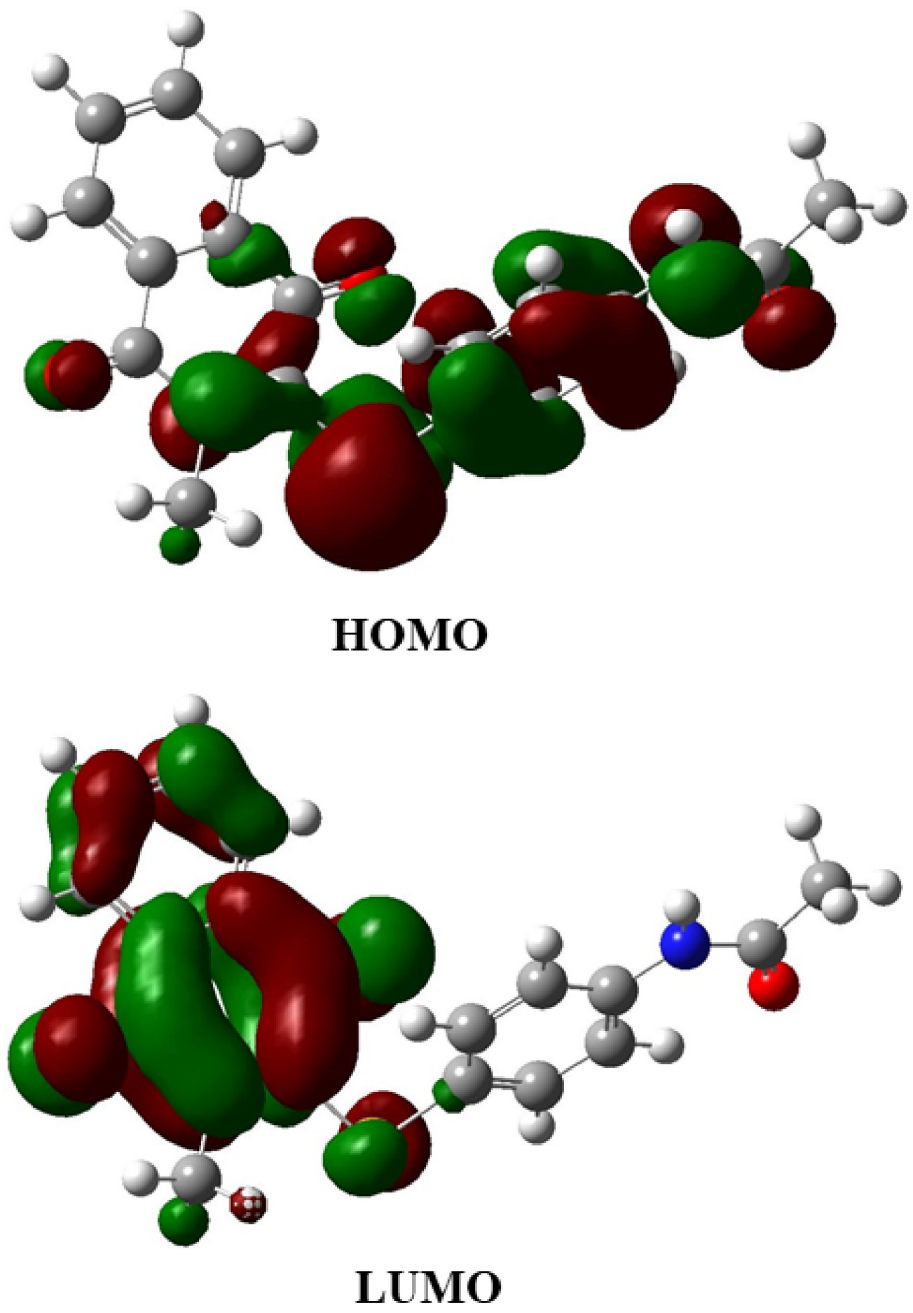
Plots of the frontier orbitals for compound **5**.

The DFT structure optimization of compound **5** was performed using the B3LYP/6-311+ G(d,p) level of theory in the gaseous phase (Figure 10). Calculations were performed to estimate the stability of the studied compound, the reactivity chemical by determining the HOMO-LUMO energy differences, the ionization potential (I), the affinity electronics (A), the electrophilicity index (ω), the chemical potential (μ), and the hardness (η) and the softness (S) of compound **5**. The determination of their optimized potential surface molecular electrostatic (MEP), to characterize the influence of the different substitution groups, has also been carried out. Finally, a study was carried out to demonstrate the rich sites and the poor sites of electrons. The visualized HOMO and LUMO of compound **5** are shown in Figure 11.

The results are presented in different, numbered tables. In addition, it seems interesting to us to start with the results obtained from the geometric parameters molecules studied. The results obtained concerning this calculation are grouped in Table 4 according to the following numbering.

Compare these theoretically calculated geometric parameters with experimental data from a crystallographic analysis [79], knowing that the relative deviation Δ for a geometric parameter X is expressed as a percentage by the relation:

$$\Delta =\frac{\left|X_{theo}-X_{exp}\right|\;}{X_{exp}}\times \;100$$

X*_theo_*: the theoretical value of the quantity X.

X*_exp_*: the experimental value of the quantity X.

Analysis of the results reported in Table 4 shows that the average deviation of the distances and angles obtained by the DFT method is on the order of less than (3%) overall, this lets us say that the different theoretical results obtained are in very good agreement with those obtained experimentally by crystallographic analysis.

#### 3.6.2. Surfaces with Molecular Electrostatic Potential (MEP)

**Figure 12:**
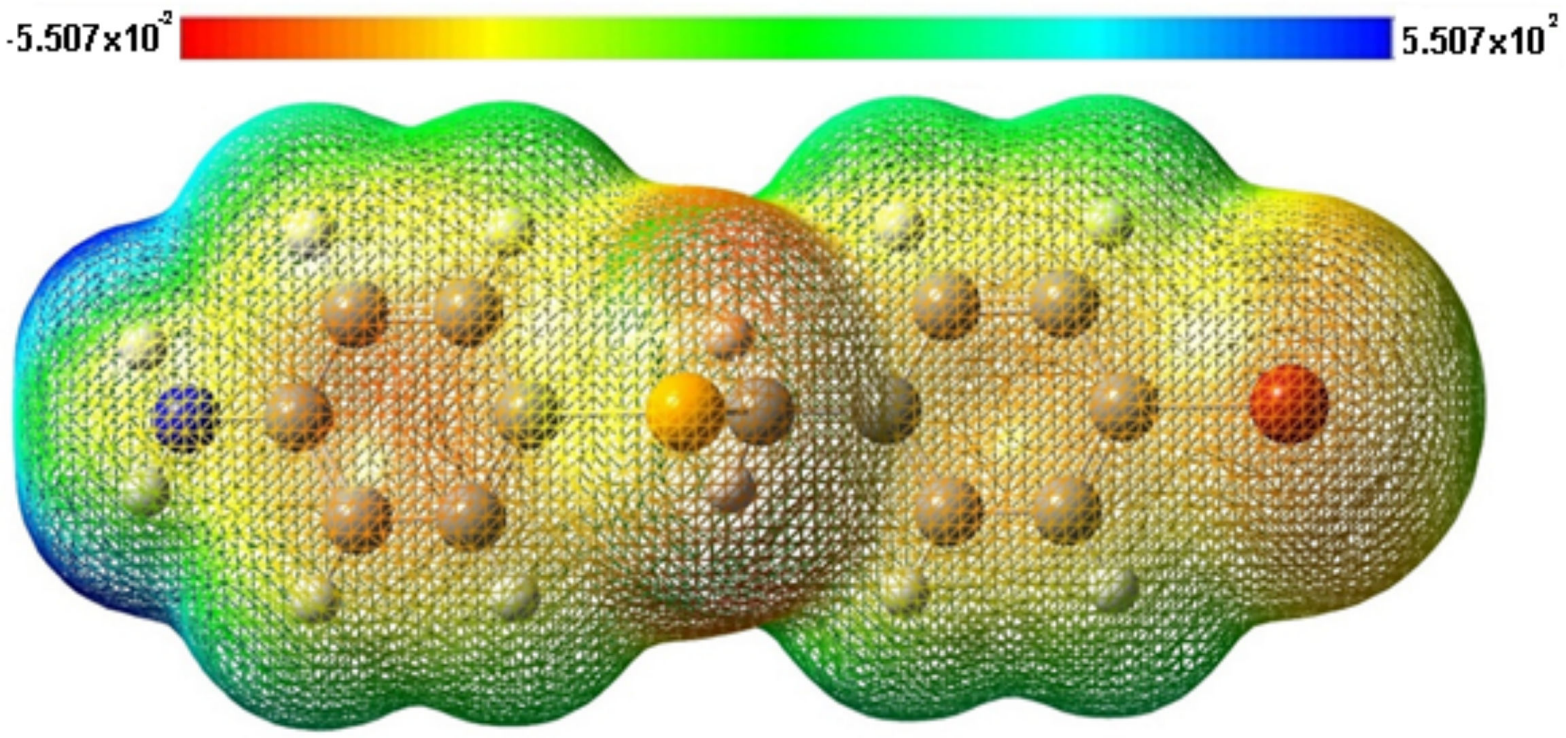
Molecular electrostatic potential (MEP) map of compound **5** calculated at the 6-311+G(d,p) level.

Molecular electrostatic potentials (MEP) are amongst the descriptors to correlate physicochemical properties with molecular structure. Therefore, MEP can differentiate sites of electrophilic attack (electron-rich areas) from nucleophilic attack (electron-poor areas). MEP are useful tools for predicting biological processes, especially sites of electrophile or nucleophile attack by a reagent. In Figure 12, the different amplitudes of the MEP are shown by different colors as follows: red < orange < yellow < green < blue [80].

The region close to the N atoms of the pyridine and imidazole ring is characterized by red and yellow and exhibits high electron density (DE). It, therefore, is attributed to a negative potential. Thus, the electrophilic attack is more likely preferred at these sites. 

#### 3.6.3. Study of Overall Global Reactivities

The HOMO-LUMO gap is an essential factor in the chemistry of quantum; it grants the characterization of molecular stability. Furthermore, frontier molecular orbitals are essential in the prediction of molecular reactivity [81,82]. Molecules with a small HOMO–LUMO energy gap could be distorted to increase this gap. The results of the descriptors calculated by DFT (Table 5) are the HOMO and LUMO energies, energy gap (Δ*E*), electronegativity (*χ*), the hardness (*η*), the chemical potential (*μ*), the global softness (*S*), the global electrophilicity index (*ω*), and the softness (*σ*), respectively, and given by the Equations (1)–(8) [83]:

*χ* = −1/2 (*E*_LUMO_ + *E*_HOMO_) (1)

*µ* = −*χ* = 1/2 (*E*_LUMO_ + *E*_HOMO_) (2)

*η* = 1/2 (*E*_LUMO_ − *E*_HOMO_) (3)

*S* = 1/2 *η* (4)

*ω* = *µ*^2^/2 *η* (5)

*σ* = 1/*η* (6)

Vertical Ionizatio Potential (VIP) = *E*_cation_ − *E*_neutral_ (7)

Vertical Electron Affinity (VEA) = *E*_neutral_ – *E*_anion_ (8)

The compound studied has a hard character because it carries a high value of chemical hardness. On the other hand, the compound carries the low value of the chemical potential followed by the high value of the electrophilicity index, hence the compound promotes its electrophilic behavior.

From a biological point of view, selenoquinone compounds (e.g., compounds **4** and **5**) demonstrated potential cytotoxicity. In theory, the Se and quinone centers complement each other, i.e., the quinone redox cycling process leads to the formation of superoxide radicals [52,53,54,55]. The latter, in turn, activates the Se center to further induce oxidation of redox-sensitive cellular compartments (e.g., enzymes and proteins) and, thus, causes cell death [52,53,54,55]. These results were in agreement with the DFT calculations in which the HOMO is mainly located on the nucleophilic centers (e.g., quinone and Se) of compound **5**, however, it is still too early to reach conclusions at this point. An unambiguous QSAR requires a diverse and huge number of compounds to get a clear understanding of the relation between the DFT calculation results and potential cytotoxicity. Ultimately, this justifies the requirements of more studies, such as additional cytotoxicity experiments and computational calculations to identify the possible parameters and the underlying structural requirements leading to cytotoxicity.

## 4. Conclusions

The structure of the novel organoselenium compound **5** was confirmed through employing the single-crystal X-ray diffraction technique. The layers that form parallel to a crystallographic axis are produced by H-bonding types N-H···O and C-H···O. The presence of relatively weak C···H, Se···N, and C···O interactions contributes to further crystal packing stabilization. The H···H interatomic contact, which contributes the most to crystal packing with a percentage contribution of 40%, is revealed by HS analysis for further noncovalent interaction exploration. The crystal packing’s three-dimensional topology was analyzed and visualized using energy framework calculations. The total interaction energy in the crystal packing is dispersive, according to the results.

Compound **5** exhibited potential cytotoxicity against the 158 N and 158 JP oligodendrocytes and decreased the ROS level at 10 μM compared to vitamin E. It also showed moderate GPx-like activity.

Our work is a theoretical contribution to the study of the structure; chemical reactivity of the studied compound, which plays an important role in biology; pharmacology; and synthesis. We carried out two parts of this research work. Excellent agreement between the calculated and experimental data was achieved. It is in this context that Frontier Molecular Orbital Theory, and, ultimately, Conceptual DFT emerged and developed. 

## Figures and Tables

**Table 1:** Structure refinement of compound **5** and crystal data.

Empirical Formula	C_19_H_15_NO_3_Se
M_r_	384.28
Temperature (K)	115
Crystal system	monoclinic
Space group	*P2_1_*
*a (Å)*	5.0878 (5)
*b (Å)*	24.111 (2)
*c (Å)*	6.6263 (7)
*a (°)*	90
*β (°)*	97.826 (3)
*γ (°)*	90
Volume (Å^3^)	805.30 (14)
Z	2
ρ_calc_ (g/cm^3^)	1.585
Μ(mm^−1^)	2.348
F(000)	388.0
Crystal size (mm^3^)	0.25 × 0.15 × 0.15
θ_min_/θ_max_ (deg)	6.206/55.106
Reflections collected	10,389
Independent reflections	3699
Data/restraints/parameters	3699/1/220
GOOF=S	1.001
R_1_ [F^2^ > 2 σ (F^2^)]	0.0211
wR_2_ (F^2^)	0.0468
ρ_max_/ρ_min_ (e.Å^−3^)	0.28/−0.20
Flack parameter	0.017 (8)

**Table 2:** Selected bond distances and angles of (**5**).

Bond Distances (Å)			
Se-C1	1.912 (3)	C4-C9	1.392 (4)
Se-C12	1.923 (3)	C5-C6	1.382 (5)
O1-C3	1.219 (4)	C6-C7	1.386 (5)
O2-C10	1.216 (3)	C7-C8	1.375 (4)
O3-C18	1.236 (3)	C8-C9	1.385 (4)
N-C15	1.418 (3)	C9-C10	1.490 (4)
N-C18	1.355 (3)	C12-C13	1.389 (4)
C1-C2	1.353 (4)	C12-C17	1.390 (4)
C1-C10	1.486 (4)	C13-C14	1.385 (4)
C2-C3	1.497 (4)	C14-C15	1.398 (4)
C2-C11	1.503 (4)	C15-C16	1.388 (4)
C3-C4	1.482 (4)	C16-C17	1.394 (4)
C4-C5	1.394 (4)	C18-C19	1.496 (4)
**Bond Angles (°)**			
C12-Se-C1	97.36 (11)	C10-C9-C4	120.6 (2)
C18-N-C15	125.9 (2)	C10-C9-C8	119.4 (2)
C2-C1-Se	121.0 (2)	C1-C10-O2	121.9 (3)
C10-C1-Se	117.46 (19)	C9-C10-O2	120.3 (3)
C10-C1-C2	121.4 (3)	C9-C10-C1	117.8 (2)
C3-C2-C1	120.5 (3)	C13-C12-Se	120.3 (2)
C11-C2-C1	123.6 (3)	C17-C12-Se	119.1 (2)
C11-C2-C3	115.9 (2)	C17-C12-C13	120.5 (2)
C2-C3-O1	119.8 (3)	C14-C13-C12	120.2 (2)
C4-C3-O1	121.4 (3)	C15-C14-C13	119.9 (2)
C4-C3-C2	118.7 (3)	C14-C15-N	121.8 (2)
C5-C4-C3	120.6 (3)	C16-C15-N	118.6 (2)
C9-C4-C3	120.0 (3)	C16-C15-C14	119.6 (2)
C9-C4-C5	119.4 (3)	C17-C16-C15	120.7 (2)
C6-C5-C4	120.2 (3)	C16-C17-C12	119.1 (3)
C7-C6-C5	119.9 (3)	N-C18-O3	122.7 (2)
C8-C7-C6	120.3 (3)	C19-C18-O3	121.2 (2)
C9-C8-C7	120.2 (3)	C19-C18-N	116.2 (2)
C8-C9-C4	120.0 (3)		

**Table 3:** Hydrogen-bond geometry (Å, °) in compound **5**.

*D*-H···*A*	*D*-H	H···*A*	*D*···*A*	*D*-H···*A*
**N-H···O(3) ^i^**	0.88	2.10	2.9413(3)	160
**C(13)-H(13)···O(2) ^ii^**	0.95	2.48	3.4259(4)	177
**C(19)-H(19A)···O(1) ^iii^**	0.98	2.57	3.5377(4)	170

Symmetry codes: (i) −1 + x, y, z; (ii) 1 + x, y, z; (iii) 2 − x,−1/2 + y, 1 − z.

**Table 4:** Geometric parameters obtained using DFT/B3LYP//6-311 + G(d,p).

Distance	X-ray	DFT	Valence Angles	X-ray	DFT
Se-C15	1.916 (3)	1.938	C8- Se- C15	97.31 (11)	99.78
Se-C12	1.924 (3)	1.931	C18- N- C22	125.7 (2)	129.25
O10-C11	1.219 (4)	1.221	C20- C15- Se	120.9 (2)	120.01
O7-C12	1.213 (3)	1.216	C7- C8- C9	121.7 (3)	121.91
O23-C22	1.237 (3)	1.217	C7- C8- Se	117.28 (19)	117.31
N-C18	1.423 (3)	1.410	C8- C9- C10	120.5 (3)	120.26
N-C22	1.355 (3)	1.380	C8- C9- C13	123.6 (3)	123.85
C8-C9	1.353 (4)	1.359	C10- C9- C13	115.9 (2)	115.88
C7-C8	1.486 (4)	1.499	O11- C10- C9	120.0 (3)	120.48
C9-C10	1.499 (4)	1.498	O11- C10- C5	121.4 (3)	120.91
C9-C13	1.502 (4)	1.501	C5- C10- C9	118.6 (3)	115.58
C5-C10	1.484 (4)	1.489	C6- C5- C18	120.7 (3)	119.77
C5-C6	1.396 (4)	1.397	C4- C5- C10	119.9 (3)	120.34
C5-C4	1.393 (4)	1.400	C4- C5- C6	119.4 (3)	119.88
C6-C1	1.377 (5)	1.391	C1- C6- C5	120.2 (3)	119.90
C1-C2	1.389 (5)	1.397	C6- C1- C2	120.1 (3)	120.17
C2-C3	1.377 (4)	1.391	C3- C2- C1	120.1 (3)	120.16
C3-C4	1.388 (4)	1.397	C2- C3- C4	120.3 (3)	119.84
C4-C7	1.493 (4)	1.491	C5- C4- C7	120.9 (2)	120.45
C15-C20	1.382 (4)	1.395	O23- C22- N	122.9 (2)	123.94
C15-C16	1.392 (4)	1.392	O23- C22- C24	121.1 (2)	121.40
C19-C20	1.388 (4)	1.388	N- C22- C24	115.9 (2)	114.65
C18-C19	1.400 (4)	1.402	C19- C18- N	121.5 (2)	117.20
C17-C18	1.383 (4)	1.401	C17- C18- N	118.4 (2)	123.53
C16-C17	1.395 (4)	1.392	C16- C15- Se	119.0 (2)	120.36
C18-C19	1.500 (4)	1.403	C20- C15- Se	120.4 (2)	120.00

**Table 5:** Global indices of the reactivity of the compound **5**.

Electronic Energy (eV)	−92,936.56
*E*_HOMO_ (eV)	−6.18
*E*_LUMO_ (eV)	−3.32
Gap, ∆*E*	2.86
Dipole moment, (Debye)	4.97
Chemical potential (eV)	−4.75
Electronegativity	4.75
Hardness	1.43
Softness	0.72
Global Softness	0.69
Electrophilicity index	7.89
Vertical Ionizatio potential (VIP)	3.40
Vertical Electron Affinity (VEA)	1.22

## Data Availability

The raw/processed data generated in this work are available upon request from the corresponding author.

## Supplementary Materials

The following supporting information can be downloaded at: https://www.mdpi.com/article/10.3390/ijms23052716/s1. Reference [84] is cited in the supplementary materials.

## References

[1] Xu J., Gong Y., Sun Y., Cai J., Liu Q., Bao J., Yang J., Zhang Z. (2020). Impact of selenium deficiency on inflammation, oxidative stress, and phagocytosis in mouse macrophages. Biol. Trace Elem. Res..

[2] Li B., Li W., Tian Y., Guo S., Qian L., Xu D., Cao N. (2020). Selenium-Alleviated Hepatocyte Necrosis and DNA Damage in Cyclophosphamide-Treated Geese by Mitigating Oxidative Stress. Biol. Trace Elem. Res..

[3] Schomburg L. (2020). The other view: The trace element selenium as a micronutrient in thyroid disease, diabetes, and beyond. Hormones.

[4] Lenardão E.J., Santi C., Sancineto L. (2018). New Frontiers in Organoselenium Compounds.

[5] Álvarez-Pérez M., Ali W., Marć M.A., Handzlik J., Domínguez-Álvarez E. (2018). Selenides and diselenides: A review of their anticancer and chemopreventive activity. Molecules.

[6] Jain V.K. (2017). An overview of organoselenium chemistry: From fundamentals to synthesis. Organoselenium Compounds in Biology and Medicine: Synthesis, Biological and Therapeutic Treatments.

[7] Barbosa N.V., Nogueira C.W., Nogara P.A., De Bem A.F., Aschner M., Rocha J.B. (2017). Organoselenium compounds as mimics of selenoproteins and thiol modifier agents. Metallomics.

[8] Duarte L.F.B., Oliveira R.L., Rodrigues K.C., Voss G.T., Godoi B., Schumacher R.F., Perin G., Wilhelm E.A., Luchese C., Alves D. (2017). Organoselenium compounds from purines: Synthesis of 6-arylselanylpurines with antioxidant and anticholinesterase activities and memory improvement effect. Bioorg. Med. Chem..

[9] Wirth T. (2015). Small organoselenium compounds: More than just glutathione peroxidase mimics. Angew. Chem. Int. Ed..

[10] Nogueira C.W., Rocha J.B. (2011). Toxicology and pharmacology of selenium: Emphasis on synthetic organoselenium compounds. Arch. Toxicol..

[11] Koyama H., Mutakin, Abdulah R., Yamazaki C., Kameo S. (2013). Selenium supplementation trials for cancer prevention and the subsequent risk of type 2 diabetes mellitus: Selenium and vitamin E cancer prevention trial and after. Nihon Eiseigaku Zasshi.

[12] Van Noord P.A., Maas M.J., Van der Tweel I., Collette C. (1993). Selenium and the risk of postmenopausal breast cancer in the DOM cohort. Breast Cancer Res. Treat..

[13] Berr C., Nicole A., Godin J., Ceballos-Picot I., Thevenin M., Dartigues J.F., Alperovitch A. (1993). Selenium and oxygen-metabolizing enzymes in elderly community residents: A pilot epidemiological study. J. Am. Geriatr. Soc..

[14] Thompson I., Kristal A., Platz E.A. (2014). Prevention of prostate cancer: Outcomes of clinical trials and future opportunities. Am. Soc. Clin. Oncol. Educ. Book.

[15] Bueno D.C., Meinerz D.F., Allebrandt J., Waczuk E.P., Dos Santos D.B., Mariano D.O., Rocha J.B. (2013). Cytotoxicity and genotoxicity evaluation of organochalcogens in human leucocytes: A comparative study between ebselen, diphenyl diselenide, and diphenyl ditelluride. BioMed Res. Int..

[16] Bhattacharya A. (2011). Methylselenocysteine: A promising antiangiogenic agent for overcoming drug delivery barriers in solid malignancies for therapeutic synergy with anticancer drugs. Expert Opin. Drug Deliv..

[17] Papp L.V., Lu J., Holmgren A., Khanna K.K. (2007). From selenium to selenoproteins: Synthesis, identity, and their role in human health. Antioxid. Redox Signal..

[18] Levander O.A., Alfthan G., Arvilommi H., Gref C.G., Huttunen J.K., Kataja M., Koivistoinen P., Pikkarainen J. (1983). Bioavailability of selenium to Finnish men as assessed by platelet glutathione peroxidase activity and other blood parameters. Am. J. Clin. Nutr..

[19] Mugesh G., Du Mont W.W., Sies H. (2001). Chemistry of biologically important synthetic organoselenium compounds. Chem. Rev..

[20] Nogueira C.W., Zeni G., Rocha J.B. (2004). Organoselenium and organotellurium compounds: Toxicology and pharmacology. Chem. Rev..

[21] Soriano-Garcia M. (2004). Organoselenium compounds as potential therapeutic and chemopreventive agents: A review. Curr. Med. Chem..

[22] Soares F.A., Farina M., Boettcher A.C., Braga A.L., Rocha J.B.T. (2005). Organic and inorganic forms of selenium inhibited differently fish (Rhamdia quelen) and rat (Rattus norvergicus albinus) delta-aminolevulinate dehydratase. Environ. Res..

[23] Plano D., Sanmartin C., Moreno E., Prior C., Calvo A., Palop J.A. (2007). Novel potent organoselenium compounds as cytotoxic agents in prostate cancer cells. Bioorg. Med. Chem. Lett..

[24] Naithani R. (2008). Organoselenium compounds in cancer chemoprevention. Mini Rev. Med. Chem..

[25] Doering M., Ba L.A., Lilienthal N., Nicco C., Scherer C., Abbas M., Zada A.A., Coriat R., Burkholz T., Wessjohann L. (2010). Synthesis and selective anticancer activity of organochalcogen based redox catalysts. J. Med. Chem..

[26] Krief A., Hevesi L. (2012). Organoselenium Chemistry I: Functional Group Transformations.

[27] Ibrahim M., Muhammad N., Naeem M., Deobald A.M., Kamdem J.P., Rocha J.B. (2015). In vitro evaluation of glutathione peroxidase (GPx)-like activity and antioxidant properties of an organoselenium compound. Toxicol. In Vitro.

[28] Luo Z., Liang L., Sheng J., Pang Y., Li J., Huang L., Li X. (2014). Synthesis and biological evaluation of a new series of ebselen derivatives as glutathione peroxidase (GPx) mimics and cholinesterase inhibitors against Alzheimer’s disease. Bioorg. Med. Chem..

[29] Giles N.M., Gutowski N.J., Giles G.I., Jacob C. (2003). Redox catalysts as sensitisers towards oxidative stress. FEBS Lett..

[30] Nascimento V., Alberto E.E., Tondo D.W., Dambrowski D., Detty M.R., Nome F., Braga A.L. (2012). GPx-Like activity of selenides and selenoxides: Experimental evidence for the involvement of hydroxy perhydroxy selenane as the active species. J. Am. Chem. Soc..

[31] Krehl S., Loewinger M., Florian S., Kipp A.P., Banning A., Wessjohann L.A., Brauer M.N., Iori R., Esworthy R.S., Chu F. (2012). Glutathione peroxidase-2 and selenium decreased inflammation and tumors in a mouse model of inflammation-associated carcinogenesis whereas sulforaphane effects differed with selenium supply. Carcinogenesis.

[32] Sarma B.K., Mugesh G. (2005). Glutathione peroxidase (GPx)-like antioxidant activity of the organoselenium drug ebselen: Unexpected complications with thiol exchange reactions. J. Am. Chem. Soc..

[33] Weglarz-Tomczak E., Tomczak J.M., Talma M., Brul S. (2020). Ebselen as a highly active inhibitor of PLProCoV2. BioRxiv.

[34] Parnham M.J., Sies H. (2013). The early research and development of ebselen. Biochem. Pharmacol..

[35] Satheeshkumar K., Mugesh G. (2011). Synthesis and antioxidant activity of peptide-based ebselen analogues. Chemistry.

[36] Wang L., Yang Z., Fu J., Yin H., Xiong K., Tan Q., Jin H., Li J., Wang T., Tang W. (2012). Ethaselen: A potent mammalian thioredoxin reductase 1 inhibitor and novel organoselenium anticancer agent. Free. Radic. Biol. Med..

[37] Ali W., Álvarez-Pérez M., Marć M.A., Salardón-Jiménez N., Handzlik J., Domínguez-Álvarez E. (2018). The anticancer and chemopreventive activity of selenocyanate-containing compounds. Curr. Pharmacol. Rep..

[38] Shaaban S., Negm A., Sobh M.A., Wessjohann L.A. (2015). Organoselenocyanates and symmetrical diselenides redox modulators: Design, synthesis and biological evaluation. Eur. J. Med. Chem..

[39] Chakraborty P., Roy S.S., Bhattacharya S. (2015). Molecular mechanism behind the synergistic activity of diphenylmethyl selenocyanate and Cisplatin against murine tumor model. Anti-Cancer Agents Med. Chem..

[40] Shaaban S., Arafat M.A., Gaffer H.E., Hamama W.S. (2014). Synthesis and anti-tumor evaluation of novel organoselenocyanates and symmetrical diselenides dyestuffs. Pharma Chem..

[41] Shaaban S., Abdel-Wahab B.F. (2016). Groebke–Blackburn–Bienaymé multicomponent reaction: Emerging chemistry for drug discovery. Mol. Divers..

[42] Shaaban S., Arafat M.A., Hamama W.S. (2014). Vistas in the domain of organo selenocyanates. ARKIVOC.

[43] Plano D., Baquedano Y., Moreno-Mateos D., Font M., Jiménez-Ruiz A., Palop J.A., Sanmartín C. (2011). Selenocyanates and diselenides: A new class of potent antileishmanial agents. Eur. J. Med. Chem..

[44] Garud D.R., Koketsu M., Ishihara H. (2007). Isoselenocyanates: A powerful tool for the synthesis of selenium-containing heterocycles. Molecules.

[45] Mecklenburg S., Shaaban S., Ba L.A., Burkholz T., Schneider T., Diesel B., Kiemer A.K., Röseler A., Becker K., Reichrath J. (2009). Exploring synthetic avenues for the effective synthesis of selenium-and tellurium-containing multifunctional redox agents. Org. Biomol. Chem..

[46] Shaaban S. (2011). Synthesis and Biological Activity of Multifunctional Sensor/Effector Catalysts. Ph.D. Thesis.

[47] Shaaban S., Diestel R., Hinkelmann B., Muthukumar Y., Verma R.P., Sasse F., Jacob C. (2012). Novel peptidomimetic compounds containing redox active chalcogens and quinones as potential anticancer agents. Eur. J. Med. Chem..

[48] Shaaban S., Sasse F., Burkholz T., Jacob C. (2014). Sulfur, selenium and tellurium pseudopeptides: Synthesis and biological evaluation. Bioorg. Med. Chem..

[49] Shaaban S., Shabana S.M., Al-Faiyz Y.S., Manolikakes G., El-Senduny F.F. (2021). Enhancing the chemosensitivity of HepG2 cells towards cisplatin by organoselenium pseudopeptides. Bioorg. Chem..

[50] El-Senduny F.F., Shabana S.M., Rösel D., Brabek J., Althagafi I., Angeloni G., Manolikakes G., Shaaban S. (2021). Urea-functionalized organoselenium compounds as promising anti-HepG2 and apoptosis-inducing agents. Future Med. Chem..

[51] Al-Janabi A.S., Zaky R., Yousef T.A., Nomi B.S., Shaaban S. (2020). Synthesis, characterization, computational simulation, biological and anticancer evaluation of Pd (II), Pt (II), Zn (II), Cd (II), and Hg (II) complexes with 2-amino-4-phenyl-5-selenocyanatothiazol ligand. J. Chin. Chem. Soc..

[52] Shaaban S., Ashmawy A.M., Negm A., Wessjohann L.A. (2019). Synthesis and biochemical studies of novel organic selenides with increased selectivity for hepatocellular carcinoma and breast adenocarcinoma. Eur. J. Med. Chem..

[53] Shaaban S., Negm A., Ibrahim E.E., Elrazak A.A. (2014). Chemotherapeutic agents for the treatment of hepatocellular carcinoma: Efficacy and mode of action. Oncol. Rev..

[54] Shaaban S., Vervandier-Fasseur D., Andreoletti P., Zarrouk A., Richard P., Negm A., Manolikakes G., Jacob C., Cherkaoui-Malki M. (2018). Cytoprotective and antioxidant properties of organic selenides for the myelin-forming cells, oligodendrocytes. Bioorg. Chem..

[55] Shaaban S., Negm A., Ashmawy A.M., Ahmed D.M., Wessjohann L.A. (2016). Combinatorial synthesis, in silico, molecular and biochemical studies of tetrazole-derived organic selenides with increased selectivity against hepatocellular carcinoma. Eur. J. Med. Chem..

[56] Shaaban S., Gaffer E.H., Jabar Y., Elmorsy S.S. (2015). Cytotoxic naphthalene based-symmetrical diselenides with increased selectivity against MCF-7 breast cancer cells. Int. J. Pharm..

[57] Shaaban S., Gaffer E.H., Alshahd M., Elmorsy S.S. (2015). Cytotoxic Symmetrical Thiazolediselenides with Increased Selectivity Against MCF-7 Breast Cancer Cells. Int. J. Res. Develp. Pharm Life Sci..

[58] Shaaban S., Zarrouk A., Vervandier-Fasseur D., Al-Faiyz Y.S., El-Sawy H., Althagafi I., Andreoletti P., Cherkaoui-Malki M. (2021). Cytoprotective organoselenium compounds for oligodendrocytes. Arab. J. Chem..

[59] Kachanov V.A., Slabko Y.O., Baranova V.O., Shilova V.E., Kaminskii A.V. (2004). Triselenium dicyanide from malononitrile and selenium dioxide. One-pot synthesis of selenocyanates. Tetrahedron Lett..

[60] Bruker (2017). APEX3 and SAINT.

[61] Sheldrick G.M. (2017). SADABS.

[62] Dolomanov O.V., Bourhis L.J., Gildea R.J., Howard J.A.K., Puschmann H. (2009). OLEX2: A complete structure solution, refinement and analysis program. J. Appl. Cryst..

[63] Sheldrick G.M. (2015). Crystal structure refinement with SHELXL. Acta Cryst..

[64] Parsons S. (2017). Determination of absolute configuration using X-ray diffraction. Tetrahedron Asymmetry.

[65] Spek A. (2009). Structure validation in chemical crystallography. Acta Crystallogr. Sect. D.

[66] Macrae C.F., Sovago I., Cottrell S.J., Galek P.T.A., McCabe P., Pidcock E., Platings M., Shields G.P., Stevens J.S., Towler M. (2020). Mercury 4.0: From visualization to analysis, design and prediction. J. Appl. Crystallogr..

[67] Frisch M.J., Trucks G.W., Schlegel H.B., Scuseria G.E., Robb M.A., Cheeseman J.R., Scalmani G., Barone V., Mennucci B., Petersson G.A. (2009). Gaussian 09.

[68] Becke A.D. (1993). Density-functional thermochemistry. III. The role of exact exchange. J. Chem. Phys..

[69] Fiolhais C., Nogueira F., Marques M. (2003). A Primer of Density Functional Theory.

[70] Grimme S., Muck-Lichtenfeld C., Antony J. (2007). Noncovalent interactions between graphene sheets and in multishell (hyper) fullerenes. J. Phys. Chem. C.

[71] Shaaban S., Ferjani H., Althagafi I., Yousef T. (2021). Crystal structure, Hirshfeld surface analysis, and DFT calculations of methyl (Z)-4-((4-((4-bromobenzyl) selanyl) phenyl) amino)-4-oxobut-2-enoate. J. Mol. Struct..

[72] Bouraoui H., Boudjada A., Hamdouni N., Mechehoud Y., Meinnel J. (2015). Crystal structure of 1,10-[selanediylbis(4,1-phenylene)]bis(2-chloroethan-1-one). Acta Cryst..

[73] Bouraoui H., Mechehoud Y., Chetioui S., Touzani R., Benmilat M.M.A., Boudjada A. (2019). Crystal structure and Hirshfeld surface analysis of (2E,20E)-1,10- [selenobis(4,1-phenylene)]bis[3 -(4-chlorophenyl)prop-2-en-1-one]. Acta Cryst..

[74] Bouraoui H., Boudjada A., Bouacida S., Mechehoud Y., Meinnel J. (2011). Bis(4-acetylphenyl) selenide. Acta Cryst..

[75] Seredyuk M., Pavlenko V.A., Znovjyak K.O., Gumienna-Kontecka E., Penkova L. (2012). Bis {4-[(3, 5-dimethyl-1H-pyrazol-4-yl) selanyl]-3, 5-dimethyl-1H-pyrazol-2-ium} chloride monohydrate. Acta Cryst..

[76] Wolff M., Grimwood D.J., McKinnon J.J., Turner M.J., Jayatilaka D., Spackman M.A. (2012). Crystal Explorer 17.5.

[77] Spackman M.A., Jayatilaka D. (2009). Hirshfeld surface analysis. CrystEngComm.

[78] Turner M.J., McKinnon J.J., Wolff S.K., Grimwood D.J., Spackman P.R., Laka D.J., Spackman M.A. (2017). CrystalExplorer17.

[79] Klocker J., Karpfen A., Wolschann P. (2003). Surprisingly regular structure–property relationships between C–O bond distances and methoxy group torsional potentials: An ab initio and density functional study. J. Mol. Struct..

[80] Zhi H., Zheng J., Chang Y., Li Q., Liao G., Wang Q., Sun P. (2015). QSAR studies on triazole derivatives as sglt inhibitors via CoMFA and CoMSIA. J. Mol. Struct..

[81] Ebersol E.E., Arslan T., Kandemirli F., Love I., Ogretir C., Saracoglu M., Umoren S.A. (2010). Theoretical studies of some sulphonamides as corrosion inhibitors for mild steel in acidic medium. Int. J. Quantum. Chem..

[82] Rezania J., Behzadi H., Shockravi A., Ehsani M., Akbarzadeh E. (2018). Synthesis and DFT calculations of some 2-aminothiazoles. J. Mol. Struct..

[83] Rajan V.K., Muraleedharan K. (2017). A computational investigation on the structure, global parameters and antioxidant capacity of a polyphenol, Gallic acid. Food Chem..

[84] Narayanankutty A., Job J.T., Narayanankutty V. (2019). Glutathione, an antioxidant tripeptide: Dual roles in Carcinogenesis and Chemoprevention. Curr. Protein Pept. Sci..

