# Supplementary material for: A Competition between Hydrogen, Stacking, and Halogen Bonding in N-(4-((3-Methyl-1,4-dioxo-1,4-dihydronaphthalen-2-yl)selanyl)phenyl)acetamide: Structure, Hirshfeld Surface Analysis, 3D Energy Framework Approach, and DFT Calculation
Source: Int J Mol Sci. 2022 Feb 28;23(5):2716. doi: 10.3390/ijms23052716 (PMC8910872; doi:10.3390/ijms23052716)
Supplement: Supplementary file 1 [file ijms-23-02716-s001.zip › ijms-1590155-supplementary.pdf]

A Competition between Hydrogen, Stacking, and Halogen Bonding in *N*-(4-((3-Methyl-1,4-dioxo-1,4-dihydronaphthalen-2-yl)selanyl)phenyl)acetamide: Structure, Hirshfeld Surface Analysis, 3D Energy Framework Approach, and DFT Calculation

Mohamed Gouda [1], Hela Ferjani [2], Hany M. Abd El-Lateef [1,3], Mai M. Khalaf [1,3], Saad Shaaban [1,4,*] and Tarek A. Yousef [2,5,*]

[1] Department of Chemistry, College of Science, King Faisal University, P.O. Box 380, Al-Ahsa 31982, Saudi Arabia; mgoudaam@kfu.edu.sa (M.G.); hmahmed@kfu.edu.sa (H.M.A.E.); mmkali@kfu.edu.sa (M.M.K.); sibrahim@kfu.edu.sa (S.S.)

[2] Department of Chemistry, College of Science, IMSIU (Imam Mohammad Ibn Saud Islamic University), Riyadh 11623, Saudi Arabia; hhferjani@imamu.edu.sa (F.H); tayousef@imamu.edu.sa (T.A.Y.)

[3] Chemistry Department, Faculty of Science, Sohag University, Sohag 82524, Egypt; hmahmed@kfu.edu.sa

[4] Department of Chemistry, Organic Chemistry Division, College of Science, P.O. Box 11432, Mansoura University, Egypt

[5] Toxic and Narcotic Drug, Forensic Medicine Department, Mansoura Laboratory, Medicolegal Organization, Ministry of Justice, Egypt

* Correspondence: sibrahim@kfu.edu.sa or dr_saad_chem@mans.edu.sa (S.S.); tayousef@imamu.edu.sa (T.A.Y.)

Material and methods

All chemicals were obtained from Sigma. Solvents were dried before use. The spectroscopic studies were measured at the "Pôle Chimie Moléculaire" de l'Université de Bourgogne (PACSMUB)". ^{1}H (300.13 MHz) and ^{13}C (75.5 MHz) NMR spectra were analyzed on Bruker 300 Avance III, spectrometers. The values of the chemical shifts (δ) are presented in parts per million relative to tetramethylsilane, using deuterated solvent as an internal standard. In the $^{1}HNMR$, DMSO-d_6 has a singlet peak in the $^{1}HNMR$ at 2.5 ppm and a residual water peak at 3.35 ppm, $CDCl_3$ has a singlet signal at 7.26 ppm. In the $^{13}CNMR$, the ^{13}C chemical shift of DMSO-d_6 is 39.52 ppm and for the $CDCl_3$ is 77.2 ppm. 2-methyl-3-bromo-l,4-naphthoquinone, 4-selenocyanatoaniline (**2**), and 4,4'-diselanediyldianiline (**3**) were synthesized according to our literature reported method [1-4].

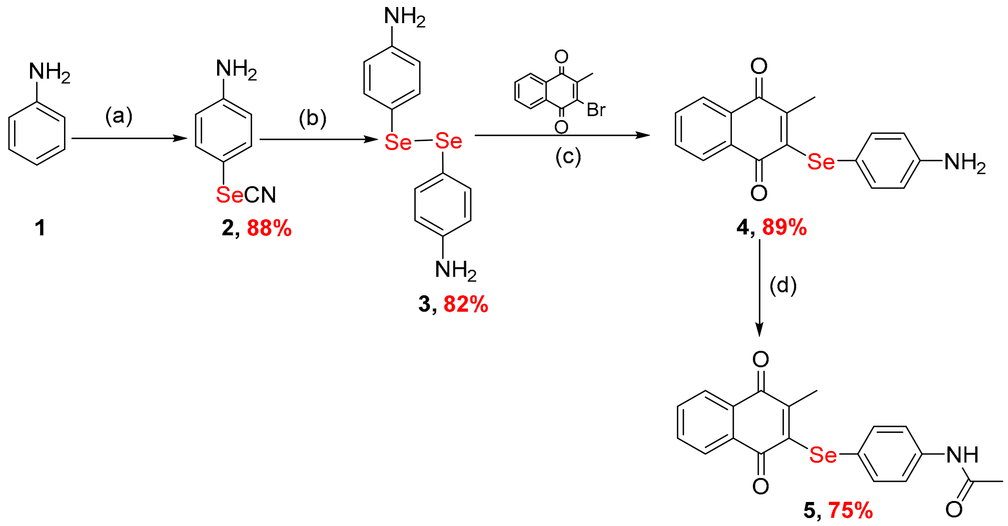

Figure S 1. Synthesis of *N*-(4-((3-methyl-1,4-dioxo-1,4-dihydronaphthalen-2-yl)selanyl)phenyl)acetamide (**5**). Reagents and conditions: (a) SeO_2 (9.6 mmol), malononitrile (4.8 mmol), DMSO (4 mL); (b) 4-selenocyanatoaniline 2 (2 mmol), NaOH (3 mmol), MeOH (15 mL); (c) 2-methyl-3-bromo-l,4-naphthoquinone (4.4 mmol), EtOAc: water (1:1), $NaBH_4$ (6 mmol), Aliquat 336 (5% mol); (d) acetic anhydride, 3 h, 50 °C.

Synthesis of 2-methyl-3-bromo-l,4-naphthoquinone *[1, 2]*

2-Methyl-1,4-naphthoquinone (11 mg, 63 mmol), anhydrous sodium acetate (1.1 mg, 34 mmol) were mixed in glacial acetic acid (50 mL). Bromine (2 mL) was then added and the mixture was allowed to settle in the dark for three days. A yellow crystalline mass formed, which was removed. The supernatant liquid was poured into 500 ml of distilled water. The formed precipitate was filtered off and recrystallized from ethanol.

Synthesis of 2-((4-Aminophenyl)selanyl)-3-methylnaphthalene-1,4-dione (4)*[3]*

Compound **4** was prepared from 4,4'-diselanediyldianiline (**3**) (344 mg, 1 mmol), 2-methyl-3-bromo-l,4-naphthoquinone (550 mg, 2.2 mmol), Aliquat 336 (45 mg, 5% mol) and sodium tetrahydridoborate (189.15 mg, 5 mmol). The progress of the product formation was followed by TLC petroleum ether: EtOAc=4:1.5, Rf=0.32, purified by column silica gel chromatography with petroleum ether: EtOAc=3:1. Brown solid; Yield: 305.27 mg (89%); mp 162–164 °C. ^{1}H NMR (300 MHz, DMSO-d_6) δ 7.97 (ddt, J=11.8, 6.9, 3.4 Hz, 2H, Ar-H), 7.87–7.77 (m, 2H, Ar-H), 7.28–7.19 (m, 2H, Ar-H), 6.55–6.44 (m, 2H, Ar-H), 5.39 (s, 2H, NH2), 1.98 (s, 3H, CH_3); ^{13}C NMR (75 MHz, $CDCl_3$) δ 181.84, 181.73, 149.10, 147.31, 147.24, 135.69, 134.08, 133.77, 131.69, 131.65, 126.34, 126.27, 114.65, 111.92, 16.50; MS (ESI): m/z=found 344.95 [M^{+}+1]; calcd. 343.01 [M^{+}]; HRMS calcd. for $C_{17}H_{13}NO_2Se$ [M^{+}+Na]: 366.00037, found 365.99930 [M^{+}+Na]. Anal. Calcd for $C_{17}H_{13}NO_2Se$ (343.01): C, 59.66; H, 3.83; N, 4.09. Found: C, 59.64; H, 3.80; N, 4.11.

Synthesis of N-(4-((3-Methyl-1,4-dioxo-1,4-dihydronaphthalen-2-yl)selanyl) phenyl)acetamide (5). *[3]*

Acetic anhydride (2.0 equivalents) was added to one equivalent of 2-((4-aminophenyl)selanyl)-3-methylnaphthalene-1,4-dione (**4**) and the reaction was heated for two hours at 60 °C. The reaction was cooled and then poured onto ice. The formed precipitated was purified by silica gel chromatography.

The progress of the product formation was followed by TLC petroleum ether: EtOAc=8:1, Rf=0.43, purified by column silica gel chromatography with petroleum ether: EtOAc=8:3. Brown solid; Yield: 288.75 mg (75%); mp 125–127 °C. ^{1}H NMR (300 MHz, DMSO-d_6) δ 8.04–7.92 (m, 2H, Ar-H), 7.89–7.78 (m, 2H, Ar-H), 7.49 (qd, J=6.7, 3.4 Hz, 4H, Ar-H), 2.07 (s, 3H, CH_3), 2.03 (s, 3H, CH_3); ^{13}C NMR (75 MHz, DMSO) δ 182.33, 181.60, 168.89, 149.50, 146.11, 139.51,

134.57, 134.01, 132.16, 126.96, 122.88, 120.29, 24.47, 17.96; MS (ESI): m/z=found 384.83 [M^+]; calcd. 385.02 [M^+]; HRMS calcd. For $C_{19}H_{15}NO_3Se$ [M^++Na]: 408.01094, found 408.00933 [M^++Na]. Anal. Calcd for $C_{19}H_{15}NO_3Se$ (384.83): C, 59.38; H, 3.93; N, 3.64. Found: C, 59.40; H, 3.95; N, 3.61.

2-((4-Aminophenyl)selanyl)-3-methylnaphthalene-1,4-dione (4)

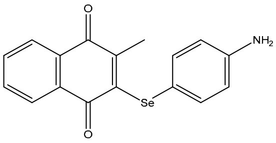

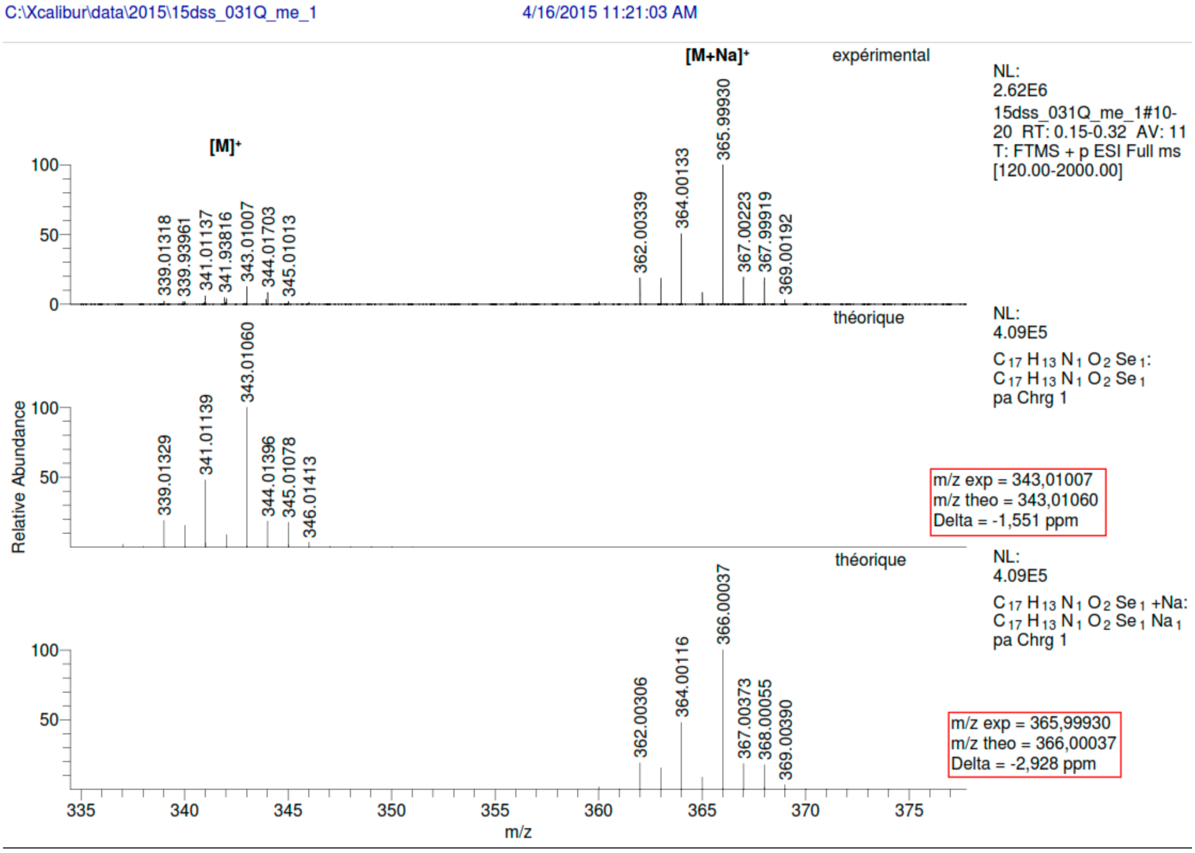

Figure S 2. HRMS of organic selenide **4**.

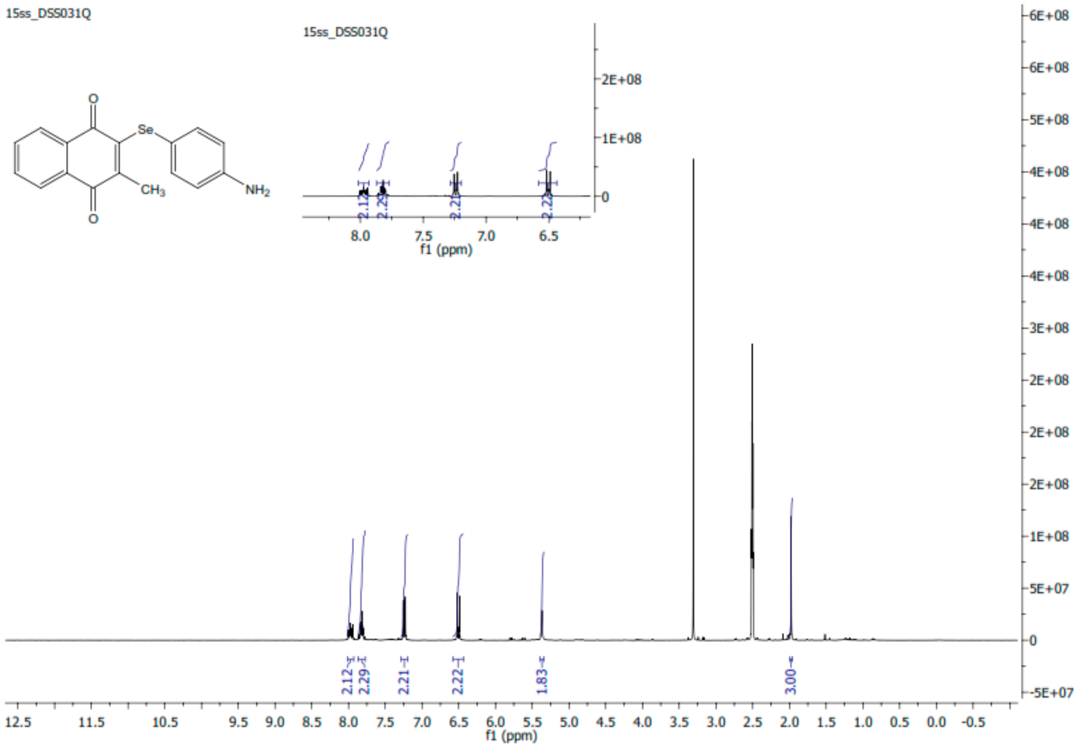

Figure S 3. ^{1}H NMR of organic selenide **4**.

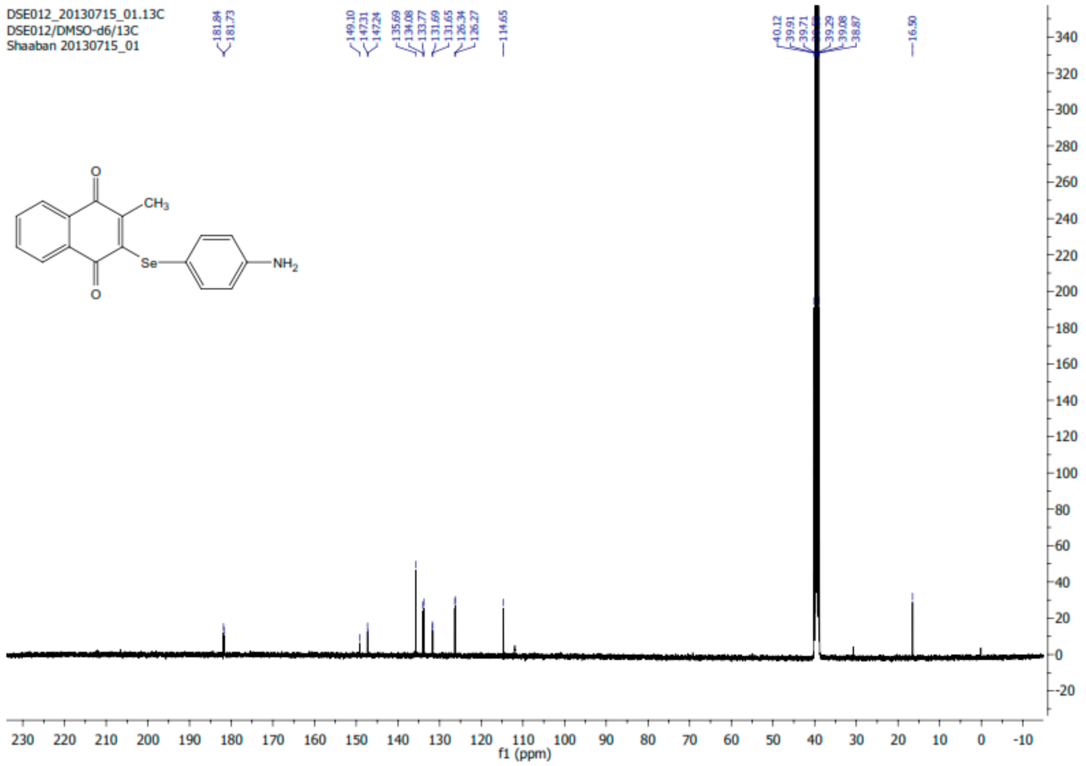

Figure S 4. ^{13}C NMR of organic selenide **4**.

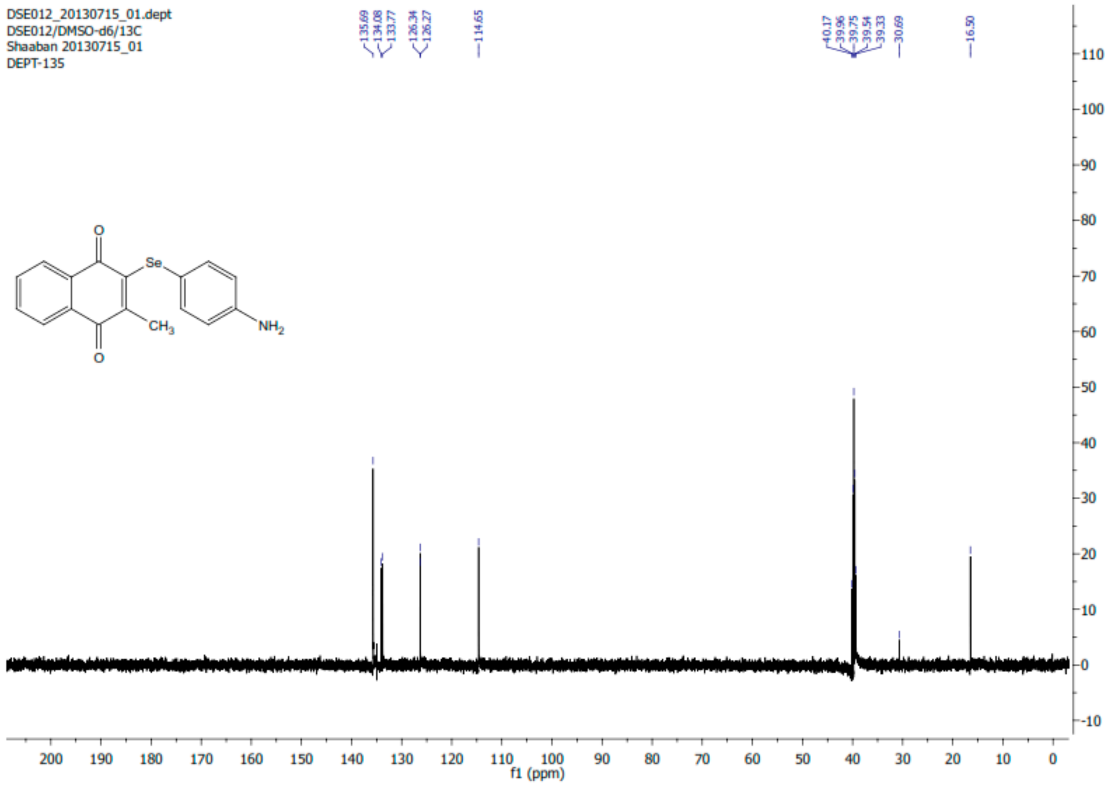

Figure S 5. DEPT135 of organic selenide **4**.

N-(4-((3-Methyl-1,4-dioxo-1,4-dihydronaphthalen-2-yl)selanyl)phenyl)acetamide (5)

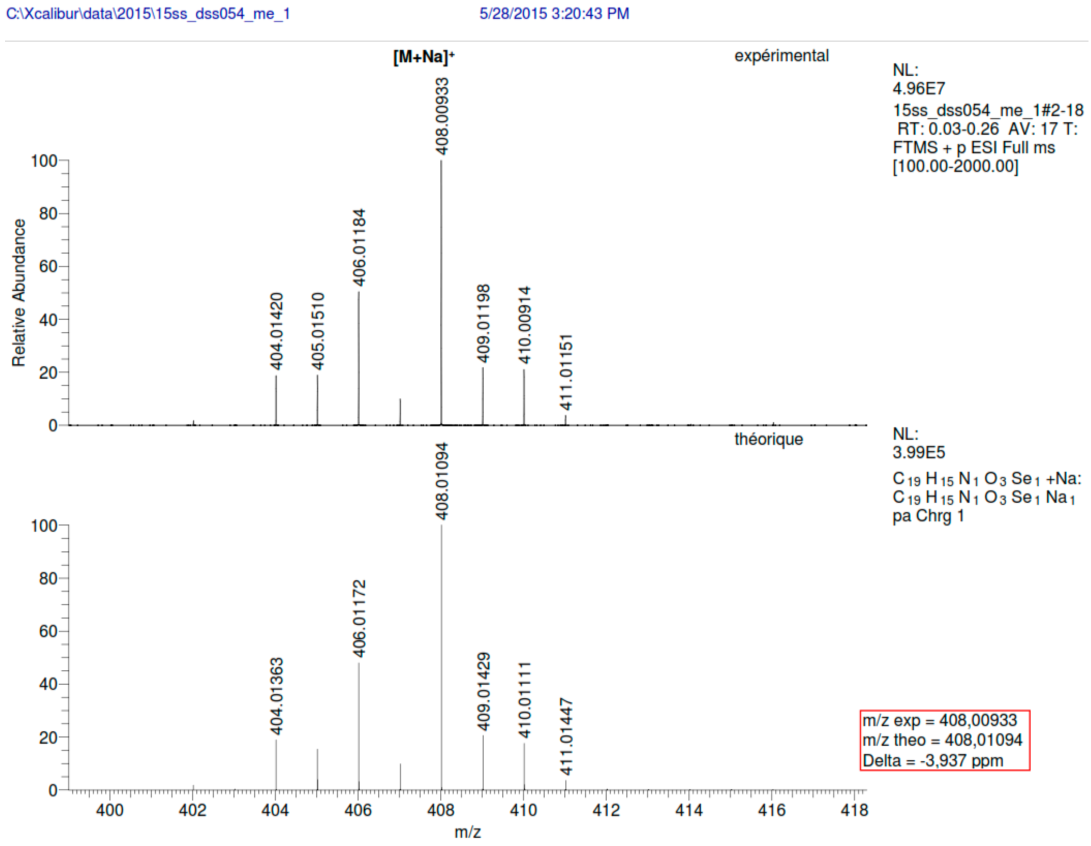

Figure S 6. HRMS of organic selenide **5**.

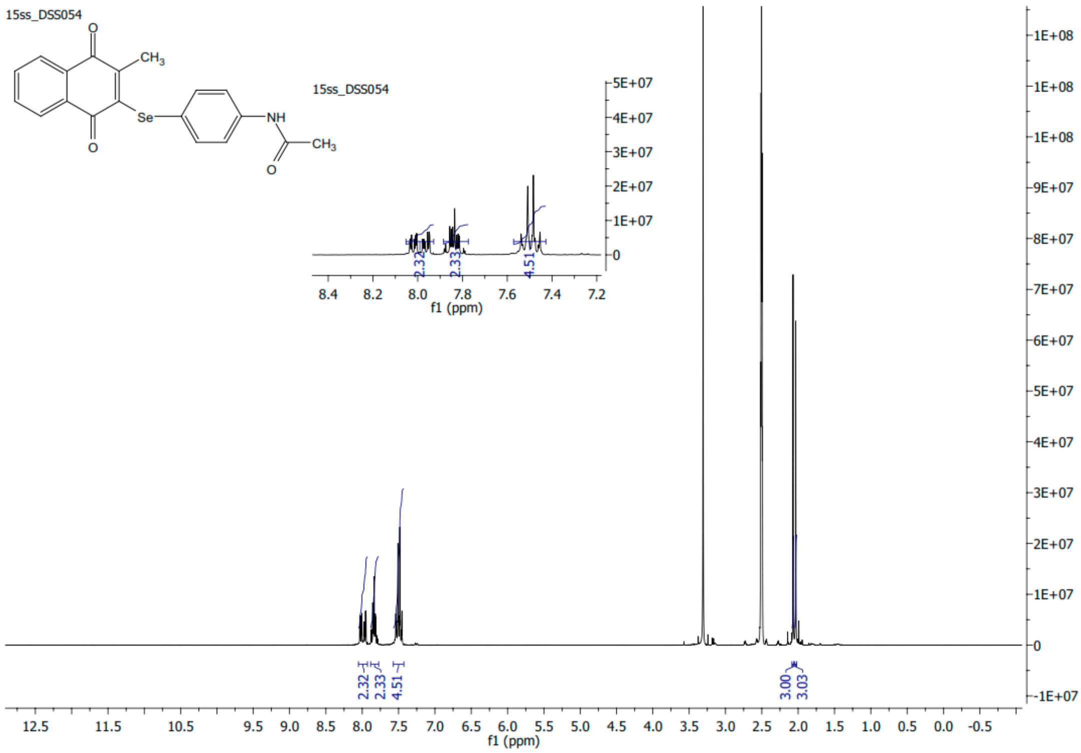

Figure S 7. ^{1}H NMR of organic selenide **5**.

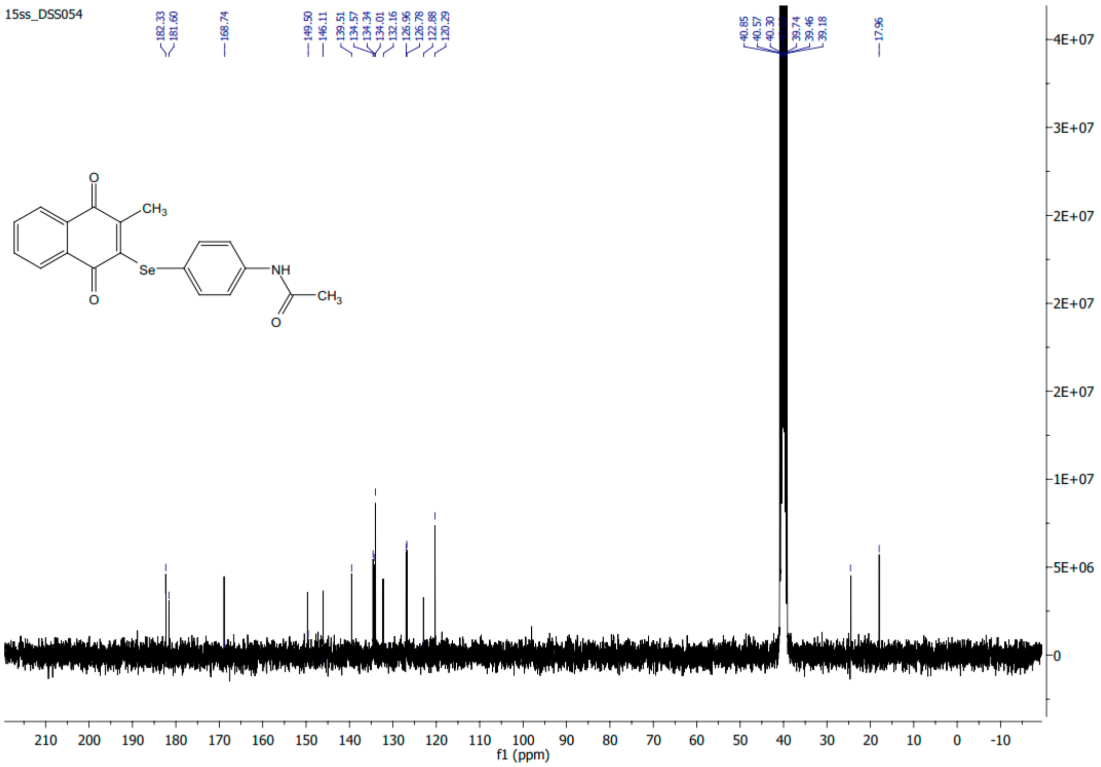

Figure S 8. ^{13}C NMR of organic selenide **5**.

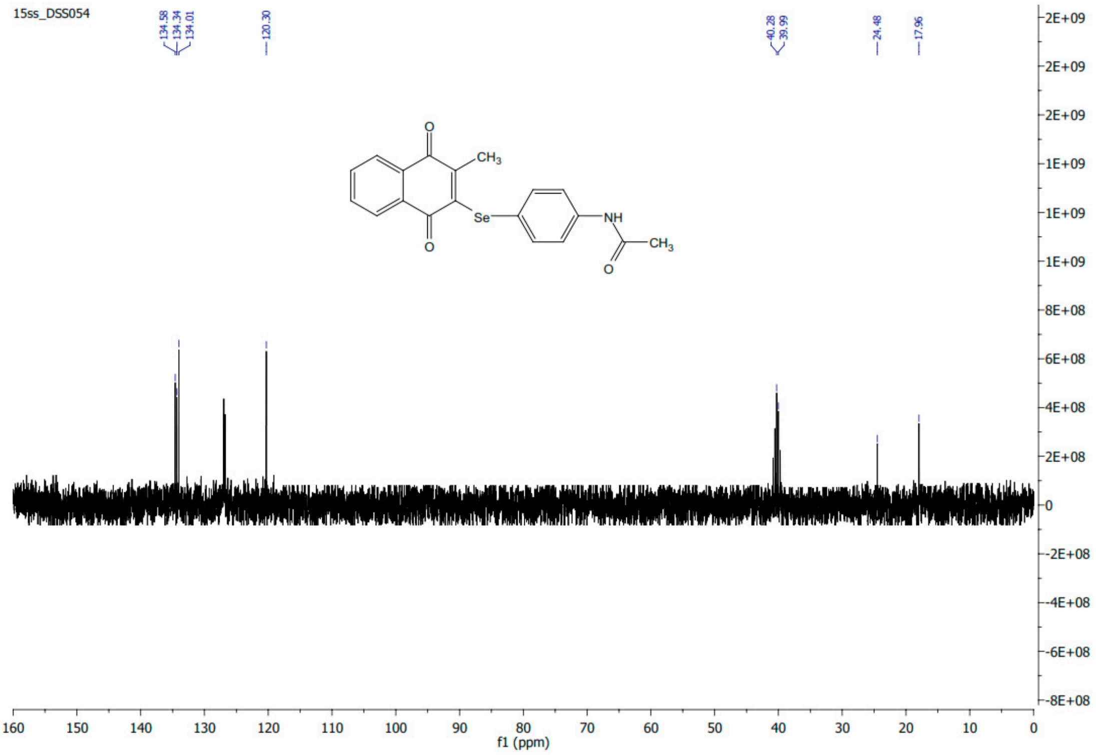

Figure S 9. DEPT135 of organic selenide **5**.

Biological assays

Cytotoxicity assay[3]

Murine oligodendrocytes (158N and 158JP) were seeded at 240,000 cells per 24-well plates and were grown using the Dulbecco's Modified Eagle Medium supplemented with penicillin antibiotics (1% v/v, streptomycin) and fetal bovine serum (5% v/v) and kept at 37 °C an atmosphere containing 5% CO_2 [3]. The concentration of 7kc [and] vitamin E and the time of treatment were chosen based on data obtained on 7kc treated 158N cells. Organic selenides **4** and **5** were prepared in DMSO and then diluted with DMEM up to 1 mM.

MTT assay

The MTT assay was carried out on 158N and 158JP cells after one day of incubation with 7kc (50µM)/different concentrations (0, 1, 10, 20, 50, and 100 µM) of the organic selenides **4** and **5** were. In live cells, the MTT is reduced to formazan by the succinate dehydrogenase mitochondrial enzyme. The microplate reader Tecan Sunrise (Tecan, Lyon, France) was used for the reading of the plates at 570 nm. The IC_{50} values and the maximum concentrations without toxicity were estimated for organic selenides **4** and **5** from the respective dose-response curves.

Table S1. Effect of the organoselenium compounds on the viability of 158N and 158JP cells.[a]

**Compounds No**	IC_{50} (µM)	
	158N	158JP
**7kc**	37	74
**4**	27	*a*
**5**	11	0.02

The cytotoxicity was measured after two days of treatment with different concentrations (0, 1, 10, 20, 50, and 100 µM) of the organic selenides using the MTT assay. The IC_{50} was estimated as the mean of two parallel experiments; 7kc was used as a positive control; [a]Means no growth inhibition was observed at the tested concentration range.

H2-DCFDA and DHE assays

ROS overproduction and $O_2^{\bullet-}$ were detected with DCF and DHE, respectively. Cultured cells were treated with trypsin re-suspended in PBS (10^6 cells/mL) and kept in the dark for a half-hour at 37°C with H2-DCFDA or DHE at 10 µM and 2 µM and cells were analyzed by flow cytometry. The green fluorescence of 2',7'- dichlorofluorescein resulting from the oxidation of H2-DCFDA was analyzed by flow cytometry and collected through a 520/10-nm bandpass filter. DHE, a non-fluorescent compound, rapidly oxidized in ethidium under the action of $O_2^{\bullet-}$ to give 2-hydroxyethidium that intercalates with DNA giving red fluorescence of ethidium collected through a 590/10-nm bandpass filter using an excitation of 488 nm and an emission wavelength of 575 nm. Flomax (Partec) or FlowJo (Tree Star Inc.) software was used for the data analysis.

Table S2. Evaluation of the effects of compounds on ROS production by H2-DCFDA assay.

	**H_2-DCFDA assay (% of control)**		
**VITAMINE E**	94 ± 5		
	**C1 (10 µM)**	**C2 (20 µM)**	**C3 (50 µM)**
**4**	700 ± 143*	854 ± 135	935 ± 307*
**5**	7 ± 2*	5 ± 1*	6 ± 1*

158N Cells were cultured in the presence of different compounds (C1=10; C2= 20; C3=50 µM); Flow cytometry technique was used to evaluate ROS levels via staining cells with H2-DCF; data are shown as mean ± SD and expressed as % control; the significance of the difference between the DMSO and compounds. Treated cells are indicated by (Mann–Whitney test; *: $P < 0.05$); vitamin E (50 µM) was used as a positive control.

Table S3. Evaluation of the effects of compounds on superoxide anions ($O_2^{\bullet-}$) production by DHE assay.

	DHE assay (% of positive cells)		
**VITAMINE E**	74± 1*		
	**C1 (10 µM)**	**C2 (20 µM)**	**C3 (50 µM)**
**4**	264±25*	406±29*	417±31*
**5**	109±12	121±1	120±4

158N Cells were grown in the presence of different compounds (C1=10; C2= 20; C3=50 µM); Flow cytometry techniques was used to estimate the $O_2^{\bullet-}$ production via staining with DHE; Data are shown as mean ± SD and expressed as % control; Significance of the difference between the DMSO and compounds. Treated cells are indicated by (Mann–Whitney test; *: $P < 0.05$); Vitamin E (50 µM) was used as a positive control.

Bleomycin-dependent DNA damage

The assay was performed according to the reported method with minor modifications **[3]**. The reaction mixture contained 0.5 mg/ml calf thymus DNA, 0.05 mg/ml bleomycin sulfate, 5 mM magnesium chloride, 50 mM ferric chloride, 2 mM tested compound. L-ascorbic acid (2 mM) was used as a positive control. The mixture was incubated at 37 °C for 1 hour. The activity of test compounds was evaluated as malondialdehyde equivalents. Thiobarbituric acid reactive substances, which arose from deoxyribose degradation of DNA were assessed. The reaction was terminated by the addition of 0.05 ml 0.1 M EDTA. The color was developed by adding 0.5 ml of 1% w/v thiobarbituric acid and 0.5 ml of 25% v/v HCl (25% v/v). The tube was capped with a screw cap and heated at 80 °C for 30 min. After cooling in ice water, the mixture was then shaken and centrifuged and the extent of DNA damage was measured by an increase in absorbance at wavelength 532 nm.

Table S4. The Bleomycin-dependent DNA damage assay.

Compd. No.	**Bleomycin-dependent DNA damage assay** (Abs.)	**% of Vitamin C**
**Vit.C**[a]	989	100
**4**	483	48.8
**5**	581	58.8

[a] ascorbic acid is used as a standard for antioxidant activity; values are means of 3 replicates; [b] not detected.

DPPH free radical scavenging activity

The hydrogen atom or electron donation ability of the corresponding compounds was measured by estimating the bleaching of the purple color of a methanolic solution of DPPH. This spectrophotometric assay uses stable DPPH· as a reagent. The sample was prepared by adding 200 μL of the organic selenides (1 μM in methanol) to 400 μl DPPH in methanol. After 30 min of incubation in the dark, the absorbance was read against a blank at 517 nm. Ascorbic acid (vitamin C) and ebselen were used as standard antioxidants (positive control). A blank sample was run without DPPH. A negative control sample was run using methanol instead of the sample. The radical scavenging activity was calculated using the following equation: I%= $(A_{blank}-A_{sample})/(A_{blank})x100$

ABTS assay

The antioxidant activity of the investigated compounds was assessed using 2,2'-azino-bis(3-ethylbenzothiazoline-6-sulphonic acid (ABTS) method. The radical cation derived from ABTS was prepared by the reaction of 60 mM ABTS solution with 0.3 M Manganese dioxide solution in 0.1 M phosphate buffer, pH 7. Then, the mixture was shaken, centrifuged, filtered, and the absorbance ($A_{control}$) of the resulting green-blue solution (ABTS radical solution) was measured at wavelength 734 nm. Then, 50 μl of 1 mg/ml test compound in phosphate-buffered methanol was added. The absorbance (A_{test}) was measured. The reduction in color intensity was expressed as % inhibition. The % inhibition for each compound is calculated from the following equation

$$\text{Inhibition\%} = (A_{control}-A_{testmple})/(A_{control})x100$$

Ascorbic acid (vitamin C) was used as a standard anti-oxidant (positive control). A blank sample was run without ABTS and using MeOH/phosphate buffer (1:1) instead of the sample. A negative control sample was run with MeOH/phosphate buffer (1:1) instead of the tested compound.

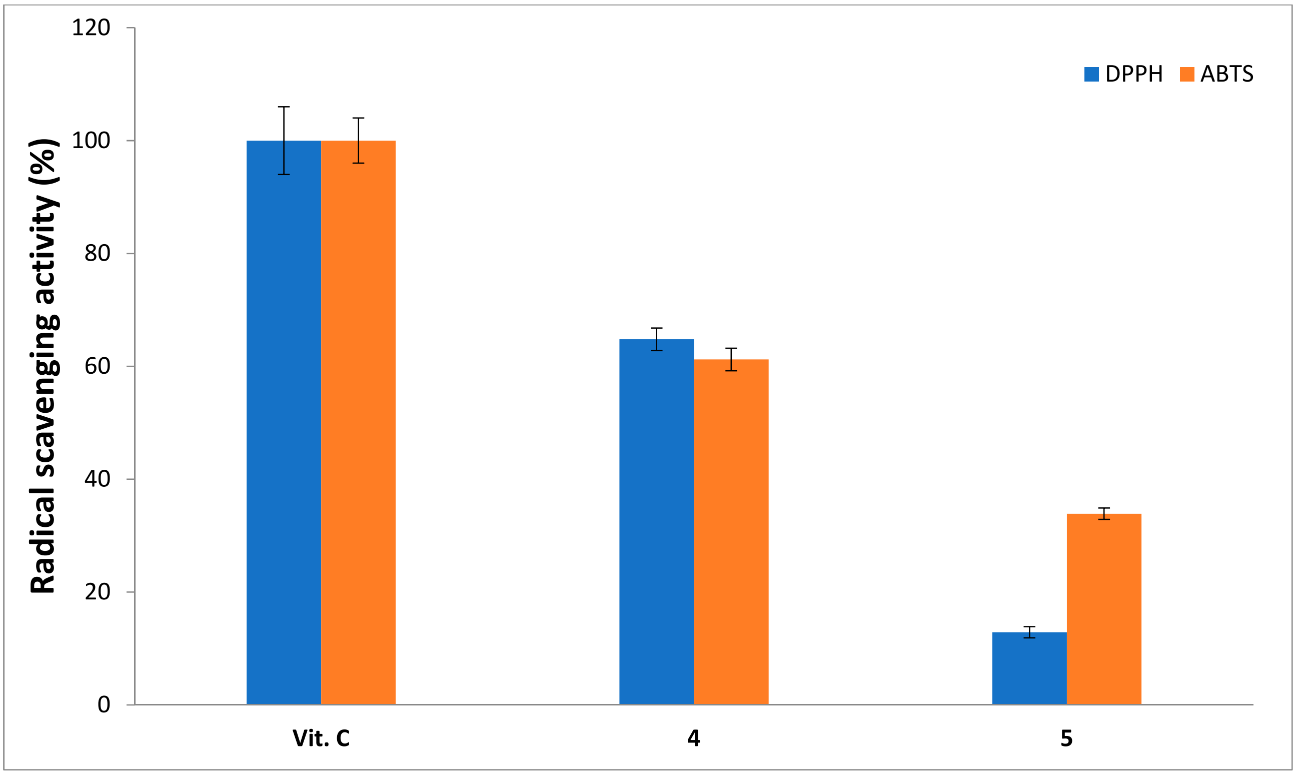

Figure S10. The DPPH and ABTS free radical scavenging chemical assays. Ascorbic acid was used as the positive control. Values are expressed as mean ± SD. Compounds were dissolved in methanol to obtain a final concentration of 1 mg/ml. 200 µL of each sample were added to 400 µl of 0.1 mM DPPH in methanol and the absorbance was read at 517 nm after 30 minutes. For the ABTS assay, the absorbance ($A_{control}$) of the resulting green-blue solution (ABTS radical solution) was measured at 734 nm after the addition of 50 µl of 1 mg/ml of the tested compound in phosphate-buffered methanol “for more details, see Material & Methods section”.

GPx -like activity

The GPx-kit was purchased from Biodiagnostic (Egypt) and used according to the reported method. The sample was prepared by adding assay buffer and NADPH reagent (0.1 ml; 24 µmol Glutathione, 4.8 µmol NADPH and12 units Glutathione reductase), and organic selenide **4** (0.01 ml, 41 µM). H_2O_2 (0.8 mM) was then added, and the absorbance was kinetically recorded every 1 min at wavelength 340 nm over three successive minutes. A_{340nm}/min was calculated. Ebselen (41 µM) was used as the positive control. For colored organoselenium compounds, their absorbance was subtracted at the used wavelength.

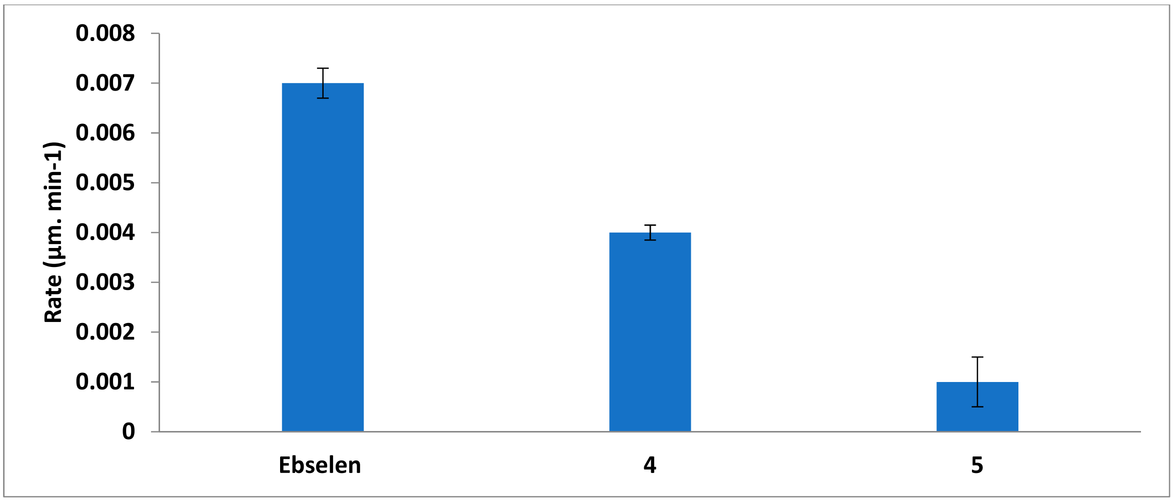

Figure S11. GPx-like activity assay (µM. min^{-1}). The reduction of H_2O_2 by GPx-like activity was tracked by oxidation of glutathione disulfide (GSSG) and NADPH to glutathione (GSH, reduced form) and NADP+, respectively. The latter was detected by a decrease in the absorbance at 340 nm.

References

1. Shaaban S, Sasse F, Burkholz T, Jacob C. Sulfur, selenium and tellurium pseudopeptides: synthesis and biological evaluation. *Bioorg. Med. Chem.* 22(14), 3610-3619 (2014).

2. Shaaban S, Arafat MA, Gaffer HE, Hamama WS. Synthesis and anti-tumor evaluation of novel organoselenocyanates and symmetrical diselenides dyestuffs . *Der Pharma Chemica*. 6, 186-193 (2014).

3. Shaaban S, Vervandier-Fasseur D, Andreoletti P *et al*. Cytoprotective and antioxidant properties of organic selenides for the myelin-forming cells, oligodendrocytes. *Bioorg. Chem.* 80, 43-56 (2018).

4. Narayanankutty A, Job JT, Narayanankutty V. Glutathione, an antioxidant tripeptide: Dual roles in Carcinogenesis and Chemoprevention. *Current Protein and Peptide Science*. 20(9), 907-917 (2019).
